# A Fully Human Engineered Bone Niche With Endogenous Osteoclastogenesis Reveals Osteoclast‐Dependent Osteomimicry in Prostate Cancer Cells

**DOI:** 10.1002/adhm.202505893

**Published:** 2026-06-11

**Authors:** Andrea Mazzoleni, Robin Dolgos, Thomas Menter, Boris Dasen, Arnaud Scherberich, Clémentine Le Magnen, Manuele G. Muraro, Ivan Martin

**Affiliations:** ^1^ Department of Biomedical Engineering University of Basel Basel Switzerland; ^2^ Department of Biomedicine University of Basel and University Hospital of Basel Basel Switzerland; ^3^ Department of Urology University Hospital Basel Basel Switzerland; ^4^ Institute of Medical Genetics and Pathology University Hospital Basel Basel Switzerland

**Keywords:** 3D in vitro model, bone metastatic niche, osteomimicry, prostate cancer bone metastasis, tumor–bone interactions

## Abstract

Bone is the predominant site of metastasis in advanced prostate cancer (PCa), yet the mechanisms governing tumor‐bone interactions remain incompletely understood, thanks in part to the scarcity of relevant models. The role played by osteoclasts in such interactions is especially obscure. A modular human three‐dimensional (3D) in vitro bone niche model was developedThe model integrates osteoblasts and osteoclasts within a mineralized scaffold, recreating an endosteal‐like microenvironment for co‐culture with PCa cell lines and patient‐derived organoids (PDOs). The engineered construct maintains osteoblastic differentiation and supports osteoclastogenesis, confirmed by lineage markers including osteocalcin, osteopontin (OPN), and tartrate‐resistant acid phosphatase (TRAP). Co‐culture with PCa cells downregulates osteoblast‐ and osteoclast‐associated genes (IBSP, OPN, TRAP) in bone cells, suggesting tumor‐mediated suppression of bone remodeling. Conversely, co‐cultured PCa cells exhibit niche‐dependent osteomimicry, characterized by upregulation of osteoblastic (SPARC, BGLAP) and osteoclastic (TRAP) markers and strongly regulated by the presence of osteoclasts. The platform also supports engraftment and proliferation of PDOs without exogenous PCa‐specific growth factors, underscoring its translational relevance. This osteoblastic‐osteoclastic niche model provides a human system that captures PCa‐bone cell interactions in a clinically relevant context, with potential utility for mechanistic and translational studies.

## Graphical abstract

A human 3D bone niche integrating osteoblasts and osteoclasts enables co‐culture with prostate cancer cell lines and patient‐derived organoids. The engineered niche (i) models aspects of reciprocal phenotypic regulation between osteoblasts and cancer cells, (ii) captures osteoclast‐enhanced osteomimicry in tumor cells, and (iii) establishes a biomimetic platform for mechanistic studies of metastatic prostate cancer cells in bone.

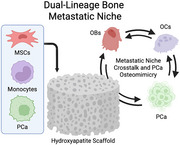

## 1 Introduction

Prostate cancer (PCa) is one of the most prevalent malignancies in men, accounting for 20% of new cancer diagnoses and representing the third leading cause of cancer‐related death among men in Europe [1]. While localized PCa is often curable, a significant portion of patients eventually progress to distant metastases, with bone being the most common site (82%) [2]. Once established in bone, metastasis‐related events profoundly compromise patient survival and quality of life, underscoring the urgent need to elucidate and therapeutically target such processes. The specialized bone niche actively supports metastatic PCa (mPCa) growth and expansion through reciprocal interactions between tumor cells and resident bone cells, disrupting the balance between osteoblastic bone formation and osteoclastic bone resorption [3]. Despite extensive research, the cellular and molecular mechanisms governing these processes remain poorly understood. Advancing our understanding of PCa‐bone interactions may pave the way for more effective or novel approaches to manage late‐stage mPCa [4].

To dissect cellular interactions in the context of mPCa, several models have been developed, predominantly relying on animal‐based systems using cell line‐ and patient‐derived xenografts, transgenic mice, or bone implant models [5]. While these systems have significantly advanced our understanding of the biology of established bone metastases, fundamental physiological differences between species limit fidelity to clinical scenarios. This disconnect is reflected in the low translational success rate of preclinical findings from animal models when tested in human clinical trials [6]. As a result, there is a pressing need for more predictive, ethically‐responsible, and 3R‐compliant platforms.

In vitro 3D metastatic models based on human cells have emerged as a powerful alternative, offering enhanced biomimicry through physiologically relevant cell‐matrix interactions while enabling modular co‐culture of stromal, immune, and cancer cell types in controlled microenvironments [7, 8]. These models hold great promise for improving mechanistic insights and developing therapeutic strategies in the context of bone metastatic PCa [9, 10, 11]. However, potentially due to the lack of important cellular components of the bone niche, existing models cannot capture pathological processes which may be critical to drive persistence of PCa cells into bone, e.g., the adaptation and acquisition of bone‐like characteristics, a phenomenon known as osteomimicry [12].

Historically, most studies exploiting PCa bone metastasis models have focused on the interactions between PCa cells and osteoblasts, reflecting the predominance of osteoblastic lesions and pathological bone formation observed in mPCa patients [13, 14, 15, 16]. However, this osteoblast‐centric approach fails to capture the reciprocal contributions of osteoclasts, whose activity not only mediates bone resorption but also fuels osteoblast activation and tumor growth. In fact, osteoclasts play essential roles in early metastatic seeding by releasing matrix‐derived growth factors and modulating osteoblast activity through paracrine signaling [17]. Excluding osteoclasts, therefore, oversimplifies the complex dynamics of the metastatic bone niche and limits mechanistic insight into the vicious cycle of remodeling [18]. When osteoclasts are included, their differentiation is often induced by high doses of exogenous RANKL, which bypasses the endogenous osteoblast–osteoclast signaling axis and may distort the niche's biology [19]. In addition to cellular interactions, the mineralized extracellular matrix is a defining feature of bone that dynamically regulates osteoblast and osteoclast activity. Its mechanical properties, particularly rigidity and mineral content, promote osteoblast differentiation and function while also modulating osteoclast adhesion, polarization, and resorptive activity, thereby shaping the dynamics of the metastatic niche [20, 21]. A mineralized, rigid surface is thus critical to provide physiologically relevant cues, which explains why many previous 3D models of PCa‐bone interactions have relied on co‐cultures with ex vivo bone tissue from mice or patients [22, 23, 24].

Here, we developed a fully human in vitro 3D model of the metastatic bone niche to test the hypothesis that osteoclasts play a critical role in shaping tumor‐bone crosstalk. Building on established differentiation protocols [25, 26, 27], the platform integrates both osteoblastic and osteoclastic precursors within a mineralized scaffold and supports controlled co‐culture with PCa cell lines and a patient‐derived organoids. By enabling osteoclastogenesis through osteoblast‐derived cues rather than exogenous RANKL, the system overcomes a significant limitation of osteoblast‐centric systems.

## 2 Results

### 2.1 Engineered Niches Recapitulate Osteoblastic and Osteoclastic Features of the Bone Microenvironment

**FIGURE 1:**
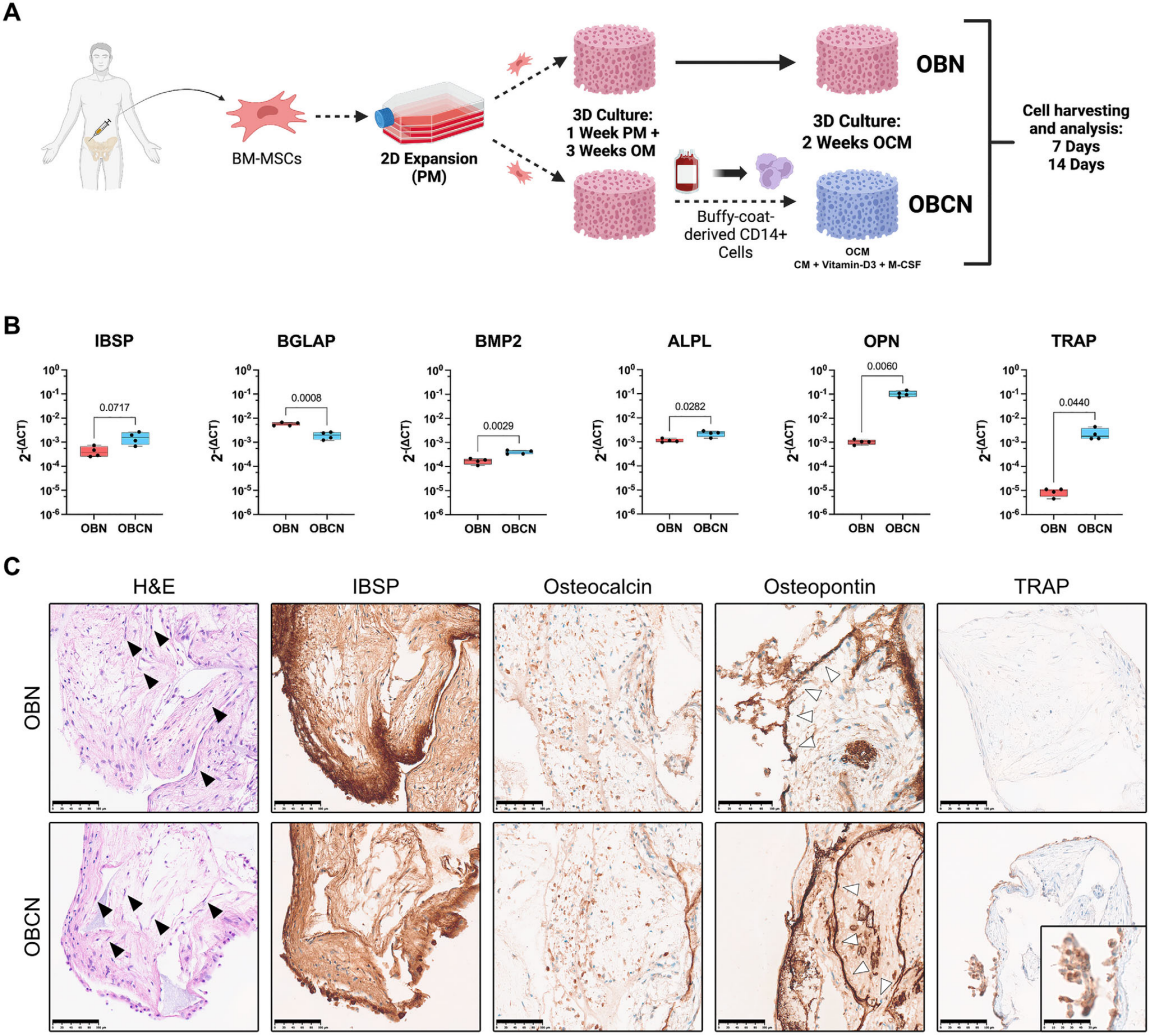
Engineered dual‐lineage human 3D bone niches integrating osteoblastic and osteoclastic cells. (A) Schematic representation of the methodology employed to engineer the niches and analyze them. Figure created with BioRender.com. (B) qRT‐PCR analysis of osteoblastic and osteoclastic markers IBSP, BGLAP, BMP2, ALPL, OPN, and TRAP in OBNs (red) and OBCNs (blue) on the mixed osteoblast and osteoclast population. Expression level was normalised to GAPDH and calculated using the 2^−ΔCT^ method. Each graph represents data from a singular donor, BM267, at the first analysis timepoint (TP1), with n=4 experimental replicates. Comparisons were done via Welch *t*‐test, with *p*‐values shown in numerical format. (C) Immunohistochemistry imaging of OBNs and OBCNs, comparing H&E staining and protein level expression of IBSP, osteocalcin, osteopontin, and TRAP (Scale bar: 100 µm). Black arrowheads in H&E images indicate cells with fusiform morphology, indicative of osteoblasts. White arrowheads in osteopontin images indicate areas adjacent to scaffold‐derived hydroxyapatite. Detailed insets in TRAP images highlight multi‐nucleated TRAP+ cells (Scale bar: 50 µm). The data are related to donor BM267 at TP1. Other donors and timepoints are shown in Figure S2.

To engineer dual‐lineage osteoblastic‐osteoclastic niches (OBCNs), we cultured human BM‐MSCs (bone marrow‐derived mesenchymal stromal cells) from different donors (n = 3) on hydroxyapatite‐based scaffolds (Engipore) for a total of 4 weeks under conditions promoting osteoblastic differentiation, followed by loading osteoclast progenitors and culturing the constructs in osteoclastic medium (OCM) for an additional 2 weeks (Figure 1A). Engipore scaffolds, mimicking the inorganic composition of bone, are known to provide osteoblastic precursors with a highly osteoconductive surface, enhancing MSC attachment [28]. Osteoclast differentiation was induced by supplementing the medium with M‐CSF and Vitamin D3, which stimulates endogenous RANKL expression in mature osteoblasts, thereby driving osteoclastogenesis through physiological osteoblast‐osteoclast signaling rather than exogenous RANKL. Osteoblastic‐only niches (OBNs) were established by omitting the osteoclastic precursors.

We first evaluated osteoblastic differentiation by gene expression analysis (qRT‐PCR; Figure 1B and Figure S1A–E). Alkaline phosphatase (ALPL), a key enzyme in matrix mineralization, showed comparable expression in OBCNs and OBNs across donors and timepoints. BGLAP, encoding osteocalcin, a late osteoblast marker, was consistently downregulated in OBCNs, with decreases ranging from 3‐fold to 11‐fold at timepoint 1 (TP1, 7 days after osteoclast seeding; *p* < 0.05) and further reduced at timepoint 2 (TP2, 14 days after osteoclast seeding; up to 9‐fold; *p* < 0.05), as expected by the absence of osteogenic stimuli. Expression of bone morphogenetic protein 2 (BMP2), a hallmark for osteoblast differentiation and bone formation, remained largely stable among donors and time points (mean < 2‐fold). In contrast, integrin‐binding sialoprotein (IBSP), involved in matrix mineralization, showed strong donor‐ and timepoint‐dependent variation: IBSP was downregulated in OBCNs from BM293 at both timepoints (*p* < 0.05), but upregulated in BM267 and BM256 at TP1 (Figure 1B and Figure S1A). Overall, gene expression showed differential expression of key markers of osteoblastic commitment, maintaining ALPL and BMP2 expression in OBCNs while downregulating BGLAP and showing donor‐specific variation for IBSP. Immunohistochemistry (IHC) analysis revealed osteocalcin‐positive cells and matrix‐associated IBSP across all conditions. Fusiform cell morphology, typical of early‐stage osteoblasts and osteoblastic progenitors, as well as the presence of osteopontin‐positive osteoblasts lining the mineral‐rich areas of the scaffolds, was evident in H&E‐stained sections both confirmed by a pathologist (Figure 1C and Figure S2A–C). Thus, our imaging findings overall confirm maintenance of osteoblastic commitment in both OBCNs and OBNs.

To evaluate osteoclastic differentiation, we examined the expression of tartrate‐resistant acid phosphatase (TRAP), a canonical osteoclast marker, alongside osteopontin (OPN), a matrix‐associated protein expressed by both osteoblasts and active osteoclasts. OPN was consistently and significantly upregulated in OBCNs across all donors and timepoints, with an increase ranging from 41 to 982‐fold (Figure 1B and Figure S1A–E). TRAP, a canonical osteoclast marker, showed similarly robust induction, with an increase exceeding 264‐fold in all OBCN donors (Figure 1B and Figure S1A–D). TRAP‐positive multinucleated cells were observed exclusively in OBCNs via IHC staining (Figure 1C and Figure S2A–C).

Together, these findings demonstrate that the OBCN platform supports the differentiation and coexistence of both osteoblasts and osteoclasts, and indicate that the presence of osteoclasts modulates the transcriptional profile of osteoblastic cells. Overall, the OBCN recapitulates phenotypic features of the bone microenvironment.

### 2.2 OBCNs Support Prostate Cancer Cell Engraftment

**FIGURE 2:**
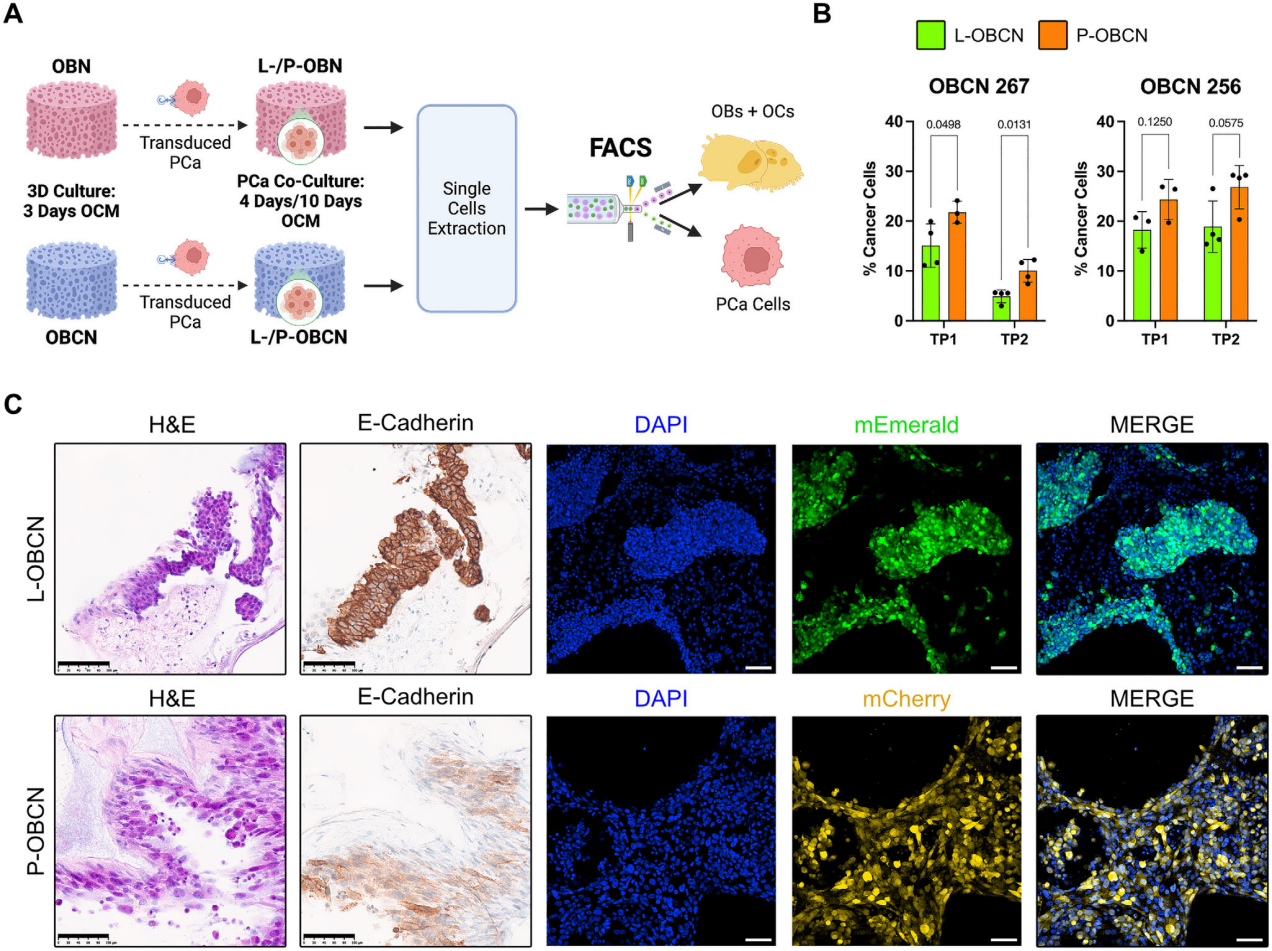
FACS and imaging reveal successful engraftment of PCa cell lines in OBCNs. (A) Schematic representation of the culture process for PCa co‐cultured niches and the procedure used for separation of osteoblasts and osteoclasts from PCa cells residing in the niches using FACS. Figure created with BioRender.com. (B) Percentage of the PCa cells retrieved from sorting of OBCNs 267 and 256 is shown for both LNCaP and PC3 lines (n=3 experimental replicates for PC3 cells in OBCN 267 and 256 at TP1 and for LNCaP cells in OBCN 256 at TP1, n=4 for all other conditions) in green and orange, respectively. The difference between LNCaP and PC3 cells extracted from niches was statistically significant for OBCN 267. Comparisons were done via Welch *t*‐test, with *p*‐values shown in numerical format. (C) Representative examples of IHC and IF imaging of L‐OBCN and P‐OBCN niches, via H&E staining, E‐Cadherin staining, and the fluorescent reporters mEmerald and mCherry (Scale bar: 100 µm). Data related to donor BM267 at TP1. Other donors and timepoints are shown in Figures S3, S4, and S5.

To evaluate whether the engineered niches support tumor cell engraftment, we seeded fluorescently labeled LNCaP (mEmerald) and PC3 (mCherry) cells into both OBNs and OBCNs, hereafter referred to as L‐OBN/OBCN and P‐OBN/OBCN, respectively. These two cell lines differ markedly in molecular features: LNCaP cells are androgen receptor–positive (AR^+^) and retain epithelial characteristics, while PC3 cells are AR^−^, display more mesenchymal‐like features, and represent an aggressive, castration‐resistant tumor phenotype. Recovered tumor cells were quantified via FACS and used for downstream analysis (Figure 2A).

A slightly higher percentage of PC3 cells was recovered from P‐OBCNs as compared to LNCaP cells in L‐OBCNs at both timepoints and donors (Figure 2B). This likely reflects intrinsic differences in growth and adaptation between the two cell lines, as PC3 cells exhibit a more aggressive, androgen‐independent phenotype, and were originally derived from bone metastases. Engraftment of epithelial tumor cells in the niches was confirmed by H&E staining and further validated by E‐Cadherin immunostaining. LNCaP and PC3 cells displayed distinct phenotypes and spatial growth patterns within the niches. PC3 cells appeared more flattened and dispersed, with irregular borders and loose cell‐cell contacts, while LNCaP cells formed compact, rounded clusters. These morphological differences were accompanied by a weaker E‐Cadherin signal in PC3 cells, consistent with their mesenchymal‐like features (Figure 2C). Whole‐mount immunofluorescence further revealed that LNCaP cells organized into larger, compact 3D aggregates, whereas PC3 cells were more diffusely distributed throughout the construct, reflecting their divergent modes of tumor growth in a bone‐like microenvironment. These observations were consistent across donors and timepoints (Figure 2C and Figures S3A–S4C). Additional evidence of PCa cell engraftment was provided by scanning electron microscopy, which indicated co‐localization of epithelial and osteoclast precursors (Figure S4).

Together, these findings demonstrate that OBCNs robustly support PCa cell engraftment.

### 2.3 PCa Cell Lines Differentially Downregulate Osteoclastic Gene Expression in OBCNs

Having established the successful engraftment of PCa cells within the bone‐like niches, we next addressed whether PCa cells modulated gene and protein expression profiles of the OBCN niche upon co‐culture, using qRT‐PCR on sorted niche cells and IHC on intact constructs.

**FIGURE 3:**
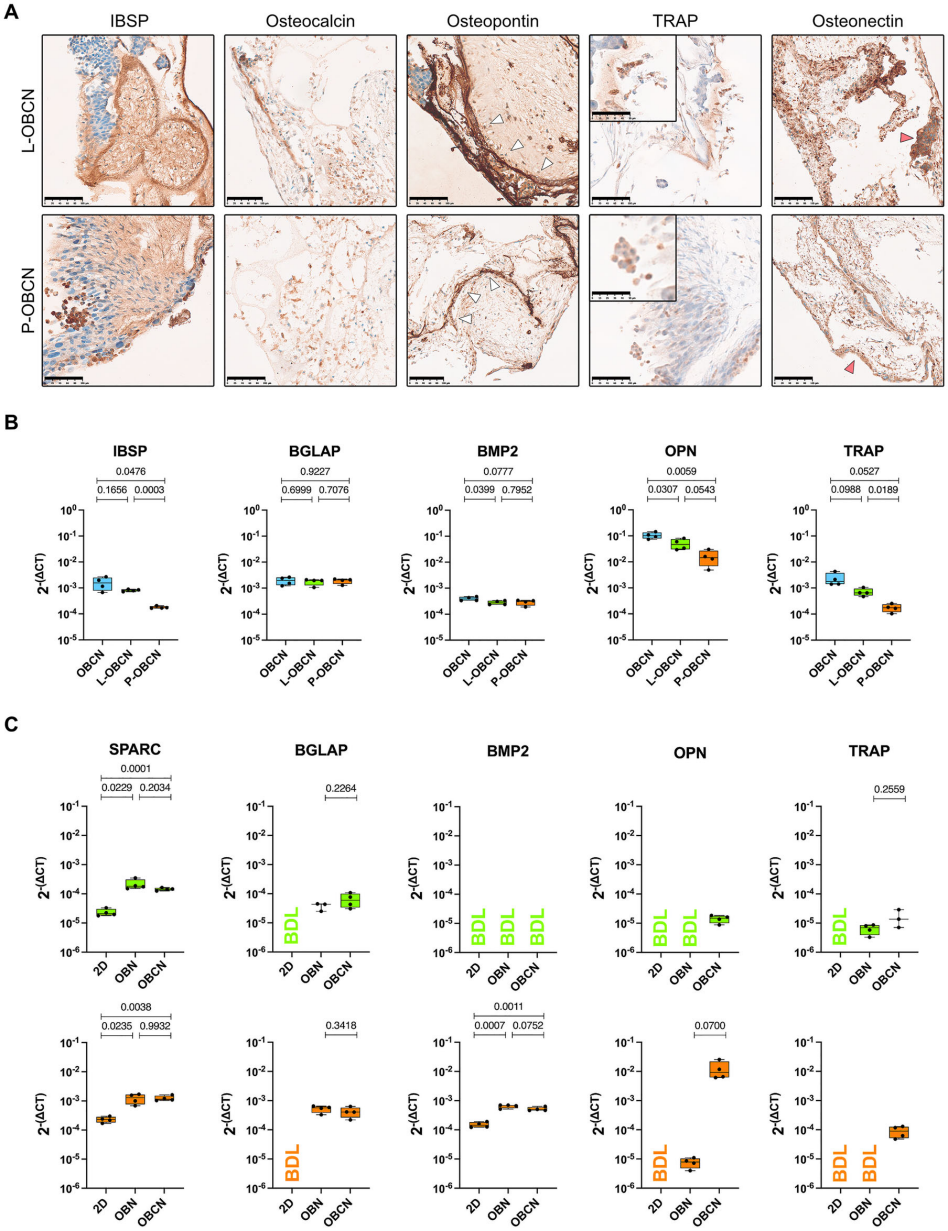
Reciprocal effects of PCa cells and engineered bone niches upon co‐culture. (A) IHC imaging comparing expression of markers IBSP, osteocalcin, osteopontin, TRAP, and Osteonectin in L‐OBCNs and P‐OBCNs (Scale bar: 100 µm). White arrowheads in osteopontin images indicate areas adjacent to scaffold‐derived hydroxyapatite. Red arrowheads show identified PCa cells expressing osteonectin. Higher‐magnification images of TRAP staining are shown, highlighting the presence of multinucleated TRAP+ cells. (Scale bar: 50 µm). (B) Gene expression analysis of bone‐related markers IBSP, BGLAP, BMP2, OPN, and TRAP in non‐tumor niche cells (FACS‐sorted). Comparisons are drawn between cells extracted from OBCNs without PCa (blue), L‐OBCNs (green), and P‐OBCNs (orange). Data for cancer‐free OBCNs are referenced in Figure 1B. (C) qRT‐PCR analysis of osteoblastic and osteoclastic markers SPARC, BGLAP, BMP2, OPN, and TRAP on PCa cells extracted from L‐OBNs and L‐OBCNs (green) or P‐OBNs and P‐OBCNs (orange). Comparisons are drawn between cells co‐cultured in the niches and the same PCa line grown in 2D under the same medium conditions. Each graph represents data from a singular donor (BM267 at TP1), with n=3 experimental replicates for conditions L‐OBN BGLAP and L‐OBCN TRAP, n=4 for all other conditions. Comparisons were done via Welch *t*‐test, with *p*‐value shown in numeric format. Conditions with undetectable gene expression have been marked with BDL (below detection level).

Histological analysis revealed no overt differences in protein expression patterns of bone‐related markers between OBCNs co‐cultured with PCa cells (L‐OBCNs and P‐OBCNs) and cancer‐cell‐free OBCNs (Figure 3A and Figure S5). IBSP retained its matrix‐associated localization, while osteocalcin (OCN) was expressed in osteoblastic cells throughout the niche. Osteopontin (OPN) remained strongly expressed in cells lining hydroxyapatite‐rich areas, and TRAP‐positive multinucleated cells were visible across all conditions. Osteonectin (SPARC), a promoter of osteoblast function and modulator of mineralization, was widely expressed in all niches, both in the extracellular matrix and in osteoblastic cells. We also noted osteonectin expression in PCa cells themselves. Thus, co‐culture with PCa cells was compatible with maintaining osteoblastic and osteoclastic populations in OBCNs.

Gene expression analysis of non‐cancerous cells isolated from OBCNs revealed dynamic modulation of bone‐related markers in both osteoblasts and osteoclasts following co‐culture with PCa cells (Figure 3B and Figure S6). IBSP expression was downregulated in both L‐ and P‐OBCNs at TP1 (4 days after seeding of PCa cells) compared to cancer‐free OBCNs, with the most pronounced decrease observed in P‐OBCNs (9‐fold lower in BM267, *p* < 0.05, Figure 3B; 4‐fold lower in BM256, n.s.; Figure S6A). By TP2 (10 days after seeding of PCa cells), IBSP expression was undetectable across all conditions, possibly due to the absence of osteogenic factors during this phase of cell culture (Figure S6B,C). BGLAP and BMP2 expression remained rather stable across most conditions, with some donor‐related variability.

For OPN and TRAP expression, we observed differential effects between L‐OBCNs and P‐OBCNs, with stronger downregulation of both markers in the latter. TRAP and OPN were consistently less expressed in P‐OBCNs relative to OBCN controls, with ∼6.5‐ and ∼13‐fold decreases in BM267 P‐OBCNs at TP1. This suppression persisted at TP2 across donors and conditions, with a reduction ranging from ∼3.5 to ∼10‐fold (Figure 3B and Figure S6A–C). A comparable trend was observed in BM256: effects were modest at TP1 (1.2‐fold, n.s., and 2.3‐fold, *p* < 0.005; Figure S6A), and more pronounced at TP2 (3.5‐fold, *p* < 0.005, and 10‐fold, *p* < 0.05; Figure S6A), where both markers were significantly reduced in P‐OBCNs. In contrast, co‐culture with LNCaP cells (L‐OBCNs) induced more modest changes in OPN and TRAP expression, which were generally not statistically significant.

Together, these results indicate that co‐culture with PCa cells alters the transcriptional profile of the bone niche, with modest and variable effects on osteoblastic genes and a more consistent suppression of osteoclastic gene expression, particularly in the presence of PC3 cells.

### 2.4 Osteoclast Presence in the Bone Niche Enhances the Osteoclastic Gene Signature of Prostate Cancer Cells

We next asked whether PCa cell phenotype is modulated by co‐culture in engineered niches. Specifically, we first assessed whether tumor cells exposed to OBNs or OBCNs acquired features of bone lineage cells, a process known as osteomimicry (Figure 3C and Figure S7A–C). Cells cultured in 2D under identical medium conditions served as controls. Among genes associated with osteoblastic differentiation and possible markers of osteomimicry, we prioritized the analysis of BGLAP, BMP2, and SPARC (osteonectin), the latter based on its dual role in bone remodeling and PCa progression [29]. IBSP was not included in this analysis due to insufficient RNA yield from the sorted tumor cell population in these specific experiments.

In 2D cultures, baseline SPARC expression in PC3 cells was approximately 10‐fold higher than in LNCaP cells (*p* < 0.005), suggesting a cell‐line‐specific response to OCM. After 4 days of co‐culture in engineered niches, SPARC expression was consistently upregulated (up to 9‐fold, *p* < 0.05) in LNCaP cells as compared to 2D controls (Figure 3C and Figure S7). In PC3 cells, SPARC was also elevated, but only in BM267‐based OBCNs (∼5.3‐fold; *p* < 0.05). No consistent differences in SPARC expression by PCa cells cultured in OBNs and OBCNs were measured. In 2D cultures, BGLAP expression was either undetectable or near the detection limit (maximum observed: 4.5 x 10^−^^6^) across both cell lines and timepoints, precluding relevant comparison with engineered niche conditions. After co‐culture in engineered niches, BGLAP was expressed at low or undetectable levels in LNCaP cells across donors and timepoints. In contrast, PC3 cells generally expressed higher levels of BGLAP across most conditions. No statistically significant differences were observed between OBNs and OBCNs using either cell line (Figure 3C and Figure S7). For 2D conditions, BMP2 expression was largely undetectable in LNCaP cells, while PC3 cells showed modest expression only at TP1 (1.5 x 10^−^^4^). In PCa co‐cultured within niches, BMP2 expression remained below detection in all LNCaP samples across donors and timepoints. In contrast, PC3 cells consistently expressed BMP2 in all 3D conditions (range: 2.7 x 10^−^^4^ to 8.0 x 10^−^^4^), suggesting that OBNs and OBCNs support or sustain BMP2 expression specifically in this cell line (Figure 3C and Figure S7). Taken together, expression patterns of these markers indicate a cell line‐specific response to the bone‐like niche in osteoclastogenic conditions: for PC3, but not LNCaP cells, it consistently induced the expression of osteoblast‐associated markers BGLAP and BMP2, while SPARC was upregulated in both cell lines in 3D, albeit with donor‐ and condition‐dependent variability.

Genes associated with osteoclastic differentiation and activity, namely OPN and TRAP, showed the most significant changes across conditions. In 2D cultures, expression was either undetectable or near the detection limit (maximum observed: 2.3 x 10^−^^6^) for both PC3 and LNCaP cells across all donors and timepoints. Upon co‐culture in engineered niches, OPN was detected in both cell lines but with markedly higher expression in PC3 cells, particularly under OBCN conditions, where it showed a ∼68 to >2700‐fold increase in expression compared to OBNs, depending on donor and timepoint (*p* < 0.05, except BM267 TP1: *p* = 0.07). In LNCaP cells, OPN expression ranged from 1.8 x 10^−^^6^ to 2.1 x 10^−^^3^, with the highest levels observed in OBCN at TP2 in both donors. TRAP expression was similarly induced in PC3 cells exclusively in OBCN conditions (range: 9.0 x 10^−^^5^–7.9 x 10^−^^4^), with no detectable expression in OBN. In contrast, LNCaP cells expressed TRAP at low but detectable levels in both OBN and OBCN (range: 6.4 x 10^−^^6^–5.9 x 10^−^^5^), with a consistent 2–6‐fold increase in OBCNs compared to OBNs. No statistically significant differences were observed between donors or timepoints, though niche‐specific trends were consistent across replicates (Figure 3C and Figure S7). Together, these findings indicate a niche‐ and cell line‐specific upregulation of osteoclast‐associated genes, with PC3 cells exhibiting strong induction of OPN and TRAP exclusively in OBCNs, while LNCaP showed lower but consistently enhanced expression of both markers in OBCNs compared to OBNs.

In summary, both PCa cell lines exhibited niche‐induced osteomimicry, with upregulation of genes associated with osteoblastic and osteoclastic differentiation. The response was largest in PC3 cells cultured in the presence of osteoclastic cells (OBCN). Our findings indicate that the inclusion of osteoclasts within the engineered bone niche has a specific and measurable effect on PCa osteomimicry.

### 2.5 PCa Patient‐Derived Organoids Successfully Engraft in OBCNs and Exhibit Osteomimicry

We next tested whether OBCNs could support and maintain patient‐derived tumor cells, extending its translational relevance. To do so, we cultured a PCa patient‐derived organoid‐to‐xenograft‐to‐organoid model (PDOXO; derived from the hormone‐naïve lung metastasis resection specimen P20‐11 [30, 31]) in hydroxyapatite scaffolds. We assessed the ability of the PDOXOs to grow on scaffolds in either OCM or organoid‐specific medium (OGM). P20‐11 cells were grown in Matrigel (MG) in the same medium conditions as the control, and their viability was assessed via CellTiter‐Glo. We then co‐cultured P20‐11 cells in the niche following the same protocol used for LNCaP and PC3 cells. OBCNs were then assessed for PCa colonization, growth, and bone‐related marker expression by immunofluorescence staining, using formalin‐fixed paraffin‐embedded (FFPE) tissues of human PCa bone metastasis samples as controls.

**FIGURE 4:**
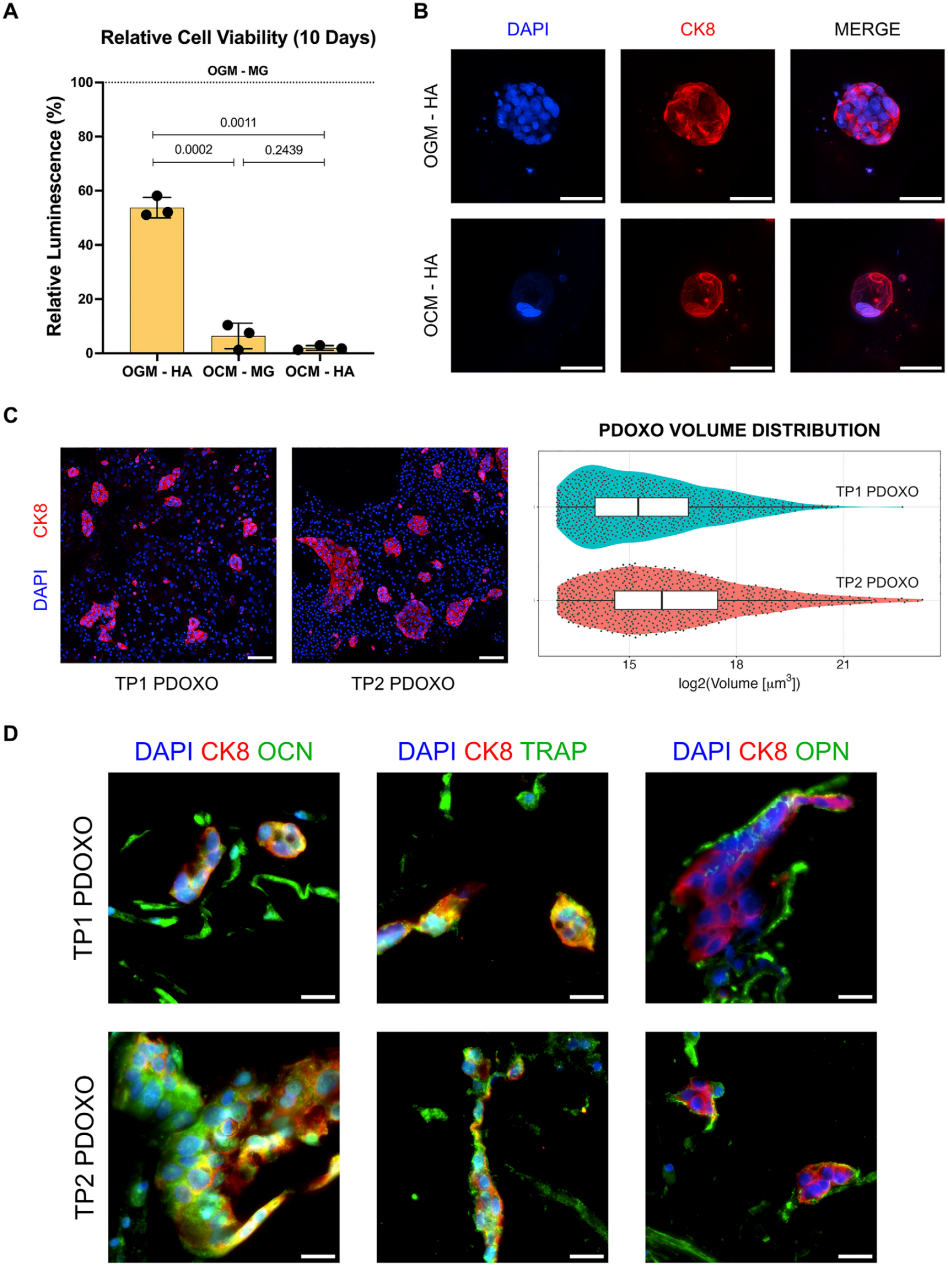
Engraftment of a patient‐derived organoid line into OBCNs and maintenance of its osteomimetic behavior. (A) Relative cell viability of a treatment naïve lung metastasis PDX‐to‐PDO line (P20‐11) cultured in organoid medium (OGM) or osteoclastic medium (OCM) in either the hydroxiapatite scaffold used for engineering OBCNs (HA), or in Matrigel (MG). Viability calculated via CellTiter‐Glo Luminescence Cell Viability Assay, according to manufacturer instructions. Luminescence measurements are taken at 10 days of culture and are normalized to the measured luminescence on cells grown in matrigel in organoid medium. Comparisons were done via Welch *t*‐test (n = 3), with *p*‐value shown in numerical format. (B) Representative immunofluorescence images of P20‐11 cells grown in HA scaffolds at 10 days of culture, showing nuclei via DAPI (blue) and Cytokeratine‐8 (red) denoting luminal epithelial cells (Scalebar: 50 µm). (C) (Left) Representative immunofluorescence images showing aggregates of the PDOXO PCa cell line, stained with DAPI (blue) and Cytokeratin‐8 (red). Images are taken at 4 days and 10 days after the start of culture in OBCNs (Scale bar: 100 µm). (Right) Distribution of CK8+ aggregate volume and their numbers for P20‐11 PDOXOs cultured for TP1 (blue) and TP2 (red). Volumes are represented as log_2_ values. Violin plots are overlaid with the median volume value. Log median difference between distributions = 0.621. Difference in median value statistically significant per Mann–Whitney U Test (p = 6.2 x 10^−10^). (D) Immunofluorescence images showing expression of bone‐related markers osteocalcin, TRAP, and osteopontin in P20‐11 cells cultured for 4 days and 10 days in the OBCNs (Scale bar: 20 µm).

At 10 days of culture, P20‐11 cells grown in OGM in hydroxyapatite were significantly more viable than the same cells cultured in OCM in either HA or Matrigel (Figure 4A), retaining 53.8 % of the viability displayed by cells grown in the control condition (OGM‐MG). Despite the difference being non‐significant, cells grown in OCM‐HA conditions were slightly more viable than those grown in OCM‐MG. Luminal PCa cells were identified by Cytokeratin‐8 (CK8) in hydroxyapatite scaffolds (Figure 4B). PDOXO cells grown in OGM‐HA conditions formed multicellular, round organoids, while they were rarely found in the scaffold and formed lower‐cell‐number aggregates when cultured in OCM. This demonstrated that OCM or HA alone was not enough to allow patient‐derived cells to adhere and be viable in the hydroxyapatite scaffolds. The PDOXOs required an organoid‐specific medium to grow in HA conditions, and did not grow in Matrigel with OCM.

PDOXO cells were co‐cultured in OBCNs and stained for CK8 at both time points. Cells grown in the niches, rather than aggregating in 3D, organoid‐like structures, adhered to the niche and spread in monolayer‐like aggregates (Videos S1 and S2). The number and volume of isolated aggregates increased slightly at 10 days (TP2) compared to 4 days (TP1; Figure 4C, *p* = 6.2 x 10^−10^), demonstrating that the engineered niches can support the short‐term culture of patient‐derived PCa cells for up to 10 days. This suggested that the microenvironment created by osteoblasts and osteoclasts in the niche provided patient‐derived PCa cells with the necessary cues for them to attach and survive, without the need for exogenous organoid‐specific stimuli.

To verify that our model reproduces the inherent osteomimetic phenotype of mPCa cells, we stained the niches for osteocalcin, TRAP, and osteopontin, the markers most strongly modulated in LNCaP and PC3 co‐culture. The CK8‐positive PDOXO cells were positive for all three markers at both time points, with membrane‐localized osteocalcin and TRAP expression and co‐expression of osteopontin observed at both time points (Figure 4D).

We next examined whether these bone‐related markers were expressed in PCa bone metastasis resections (Figure S8). In all bone metastasis samples, CK8+ cells consistently expressed osteocalcin and TRAP. Osteopontin expression was only detected in one sample, while CK8‐positive cells were adjacent to, but did not overlap with, osteopontin‐positive areas in the two other samples.

These findings offer a proof‐of‐principle that OBCNs can host and maintain PCa patient‐derived organoids in vitro without the need for growth factors comprised in the organoid‐specific medium. Furthermore, patient‐derived PCa cells express bone‐related markers within the niche, recapitulating the osteomimicry phenotype observed in both immortalized cell lines and patient samples.

## 3 Discussion

We report the engineering of a dual‐lineage (i.e., osteoblastic and osteoclastic) bone‐mimicking niche supporting engraftment of PCa cell lines and patient‐derived organoids. The fully human model enables the identification of reciprocal phenotypic changes in co‐cultured tumor and bone cells, as well as osteoclast‐regulated osteomimicry in PCa cells.

A key innovation of our approach is the integration of osteoclasts and their derivation, thanks to co‐culture with osteoblasts. While osteoclasts are often considered secondary to the osteoblastic predominance of metastatic lesions, they play essential roles in initiating niche remodeling, releasing matrix‐derived growth factors, and modulating osteoblast activity [13, 14, 15]. Previous in vitro systems have either entirely excluded osteoclasts or forced their differentiation with exogenous RANKL, thereby bypassing endogenous signaling and potentially distorting niche biology. By restoring the osteoblast‐osteoclast axis, our model captures physiological homeostatic processes, as a basis to investigate how osteoclasts contribute to the metastatic bone microenvironment.

The successful differentiation of both osteoblastic and osteoclastic lineages within the OBCNs was obtained through a sequential, modular approach used in constructing the niches. Gene and protein expression of canonical osteoblastic markers (IBSP, BGLAP, BMP2, ALPL) confirmed osteoblastic maturation across donors, despite some variability. ALPL and BMP2 were maintained, while BGLAP was consistently downregulated, and IBSP showed donor‐specific modulation at the gene expression level, while remaining consistently expressed in all conditions at the protein level. This discrepancy in gene expression of IBSP may be due to inherent biological differences in the activation kinetics of IBSP across the various donors, during osteogenic differentiation. This pattern mirrors in vivo bone remodeling compartments, where osteoblast activity is preserved, but terminal maturation is suppressed during active resorption. Osteoclast‐derived factors such as SEMA4D and S1P have been reported to inhibit osteoblast maturation under these conditions [32], suggesting that similar mechanisms may underlie the transcriptional modulation observed in our dual‐lineage niches. Osteoclastogenesis was confirmed by TRAP upregulation and the presence of multinucleated TRAP^+^ cells, consistent with Vitamin‐D3‐driven, osteoblast‐mediated RANKL induction [33, 34]. OPN transcripts were also enriched in OBCNs, aligning with its role in osteoclast‐mediated bone resorption [35, 36].

Both LNCaP and PC3 cells modulated the bone‐remodeling program of the OBCNs, with consistent downregulation of osteoclastic markers (OPN, TRAP) and selected osteogenic genes, particularly pronounced in co‐cultures with PC3 cells. This contrasts with in vivo models that report osteoclast activation by PC3 cells in metastatic lesions [18, 37], suggesting context‐dependent suppression during early‐stage adaptation. The more robust downregulation of osteoclastic markers could point to an in vitro simulation of an early‐stage response representative of in vivo osteoblastic metastases from PCa [3]. Still, longer co‐culture times and functional assessment of bone matrix turnover will be required to confirm homeostatic disruption in favour of osteoblastic bone deposition. Co‐culture in OBCNs induced a robust osteomimicry response in tumor cells, with upregulation of both osteoblastic (SPARC, BGLAP) and osteoclastic (OPN, TRAP) markers compared to 2D culture, consistent with reports linking these phenotypes to metastatic adaptation and progression [38, 39, 40, 41]. This dual osteoblastic–osteoclastic mimicry was more marked in PC3 cells, consistent with their bone‐metastatic origin, and underscores the reciprocal, cell‐line‐specific interactions between PCa cells and the human bone niche. Of note, LNCaP cells gained TRAP expression in OBNs, a trait that was not shared by PC3 cells. LNCaP cells express functionally active RANK [42], which could lead to TRAP activation after production of RANKL by mature osteoblasts in OBNs. PC3 cells also express functional RANK [43], highlighting a differential osteomimetic behavior which warrants further investigation. We hypothesize that the intense crosstalk between the androgen receptor (AR) and NF‐kB [44], an important transcription factor regulating osteoclastogenesis, might explain such a difference, with one of the main distinctions between LNCaP cells (AR+) and PC3 cells (AR‐) being their AR expression.

To investigate whether osteoclasts specifically modulate the osteomimicry response, we compared PCa cells cultured in osteoblast‐only niches (OBNs) vs. osteoblast–osteoclast niches (OBCNs). While both niche types induced similar levels of osteoblastic markers in PCa, only OBCNs consistently drove higher expression of the osteoclastic markers OPN and TRAP. This indicates that osteoclasts enhance the osteoclastic component of the mimicry program, consistent with prior reports linking osteoclast activity to increased OPN expression in cancer cells [45].

The OBCN model also supported the engraftment and maintenance of a PCa PDOXO model initially generated from a lung metastasis. Colonization of luminal PCa cells was already evident at 4 days (TP1), when single PDOXO cells had formed large aggregates attached to the scaffold, indicating successful adhesion, cell‐cell association, and potential growth. By 10 days (TP2), the median aggregate volume had further increased. The lack of PDOXO cell growth and self‐organization in OCM culture in either matrigel or hydroxyapatite conditions suggests that the presence of osteoblasts, osteoclasts, and the microenvironment they create provide PCa cells with factors, usually supplemented by organoid‐specific medium components, necessary for their survival (Figure 4A,B, OGM). The novelty of the finding is underscored by the low success rates of PCa organoid cultures [46] and the very limited number of studies achieving co‐culture of patient‐derived PCa cells in 3D mineralized bone‐like scaffolds [47, 48, 49]. Moreover, none of these prior studies combined patient‐derived PCa cells with an endogenous osteoclastogenesis driven by osteoblasts, as implemented here. Future studies should investigate which ECM‐ or cell‐derived factors allow for the growth of patient‐derived PCa cells in the niches.

The OBCN model thus represents a human‐derived system that captures key features of the endosteal niche, reciprocal molecular modulation between tumor and bone cells, and supports patient‐derived material. Moreover, its design allows for modular integration and removal of specific niche components. Considering our previously engineered microenvironments using the same scaffold, including perivascular components and supporting hematopoietic stem and progenitor cell culture [26, 27], we envision enhancing our model by integrating multiple niche types into a modular, compartmentalized bone metastasis platform. This combination provides a foundation for studying targeted dissection of niche‐specific mechanisms and testing of therapies that disrupt the spatial dependencies driving metastatic PCa [50, 51, 52]. The system could also support personalised drug testing in patient‐specific contexts.

Despite the evidence of differentiated osteoclasts in our system, the absence of direct functional validation represents a limitation of the current work. While multinucleated TRAP^+^ cells were identified within the niches and a previous study using a similar model providing proof of their functionality, no evidence of active resorption of hydroxyapatite or cell‐derived ECM was collected in the present work, leaving the question of their functionality open in this specific context. Future studies should incorporate functional assays of bone matrix turnover, such as collagen crosslink release (CTX‐I/NTX‐I) or phosphate quantification, to confirm a resorptive activity and distinguish osteoclast‐mediated degradation from passive scaffold dissolution. Another key limitation of the current configuration is the short co‐culture window with cancer cells, here limited to 10 days. Longer co‐culture times would further enable assessment of how the presence of cancer cells shifts the balance between matrix deposition and resorption. This duration was chosen based on prior observations on osteoclast differentiation and activity in 3D hydroxyapatite‐based niches, where peaking of osteoclast activity, measured by serum levels of TRAP5b, NTX‐I, and phosphates, was recorded at 13 days of culture [25]. This design ensures that molecular changes reflect interactions with a niche containing differentiated osteoclasts, but also limits the study to early stages of tumor‐bone adaptation, potentially explaining why transcriptional modulation of osteoblastic and osteoclastic markers (e.g., IBSP, TRAP, OPN) was not mirrored at the protein level. Importantly, the 14‐days osteoclastic phase was a deliberate experimental choice, not a reflection of osteoclast exhaustion or death within our system. Recent work has highlighted that long‐term retention of functional osteoclasts can be achieved through cycles of fission of mature osteoclasts and subsequent fusion of the quiescent osteomorph progeny [53]. Future iterations of the OBCN model should therefore aim to achieve longer co‐culture durations, which would also open the possibility of studying the role of osteomorphs in the vicious metastatic cycle as modeled in vitro. Another enriching exploration would involve using multiplexed protein profiling imaging techniques, such as Imaging Mass Cytometry, to understand the spatial relationship between tumor cells and bone‐marker expressing niche cells, and how these relationships are shaped by osteoclasts, something that cannot be accomplished with our current imaging framework. Moreover, testing a larger panel of patient‐derived organoids from different metastatic sites will be necessary to determine how broadly the model applies across disease subtypes. Finally, introducing scaffolds with tunable stiffness and mineral composition will extend the model's reach to the biomechanical heterogeneity of bone, offering new opportunities to explore how mechanical and biochemical signals converge to drive disease progression.

## 4 Conclusion

This study presents a fully human, engineered mPCa model in bone and supports co‐culture with PCa cells from immortalized lines and patient‐derived organoid models. The model reproduces key early features of metastatic establishment, including alterations in the bone microenvironment phenotype and the induction of dual osteoblastic‐osteoclastic osteomimicry in tumor cells. Its compatibility with patient‐derived material highlights potential applications in personalized drug testing and mechanistic studies of tumor‐niche interactions. With further refinement, this platform could help elucidate the cellular and molecular mechanisms driving metastatic PCa adaptation to bone. Finally, it could support the development of niche‐targeted therapeutic strategies that bridge tissue engineering, oncology, and personalized medicine.

## 5 Methods

### 5.1 Cell Culture Media

Complete Medium (CM) consisted of Minimum Essential Medium Alpha (aMEM) supplemented with fetal bovine serum (FBS; same lot across all experiments), GlutaMAX, Penicillin‐Streptomicin, HEPES, Sodium Pyruvate (detailed composition provided in Supporting Information file). Unless otherwise stated, all reagents were used at working concentrations.

Proliferative Medium (PM) was prepared by supplementing CM with 5 ng/mL human fibroblast growth factor 2 (FGF2). Osteoblastic Medium (OM) was prepared by supplementing CM with 0.1 mm ascorbic acid, 10 nm dexamethasone, and 10 mm beta‐glycerophosphate to promote osteogenic differentiation. Osteoclastic Medium (OCM) was composed of CM supplemented with 10 nm 1,25‐dihydroxyvitamin D3 (Vitamin D3) and 25 ng/mL human macrophage colony‐stimulating factor (detailed composition provided in Supporting Information file). This formulation is based on established osteoclastogenesis‐inducing conditions and leverages Vitamin D3's role in upregulating RANKL expression in osteoblasts [25].

### 5.2 Cell and Tissue Sources and Cell Expansion

Human bone marrow mesenchymal stromal cells (BM‐MSCs) were isolated from bone marrow aspirates obtained during routine orthopedic surgery involving iliac crest exposure, as previously described [54]. BM‐MSCs from three independent donors were used in this study: BM267, BM256, and BM293. Cells were expanded in 2D culture by seeding at a density of 5 x 10^3^ cells/cm^2^ in PM, with medium changes twice per week. Cells were passaged upon reaching 80% confluence. BM‐MSCs were either used fresh or cryopreserved at passage 2 (P2); for all experiments, cells were used at passage 2 or 3 (P2–P3).

Human CD14+ monocytes were obtained from peripheral blood collected through routine blood donations at the Swiss Red Cross (SRC) Blood Donation Center of Basel (University Hospital of Basel). Peripheral blood mononuclear cells (PBMCs) were isolated by density gradient centrifugation over Ficoll‐Paque PLUS at 400×g for 25 min at room temperature without brakes. CD14^+^ cells were subsequently purified from the PBMC fraction using magnetic‐activated cell sorting (MACS) with CD14 MicroBeads, LS columns, and a QuadroMACS Separator, according to the manufacturer's protocol.

PCa cell lines LNCaP (AR+ HSPC‐like) and PC3 (AR− CRPC‐like, bone‐derived) were cultured in standard 2D conditions by seeding at a density of 5 x 10^3^ cells/cm^2^ in CM. Medium was changed twice per week, and cells were passaged when they reached 80% confluence. LNCaP and PC3 cells were stably labeled with mEmerald and mCherry fluorescent reporters, respectively (Section S1 for full protocol).

Patient‐derived organoids (P20‐11 PDOs) were established from a treatment‐naïve lung metastasis specimen, previously described [30]. The donor of P20‐11 cells had a PSA level of 96.80 µg/l at the time of diagnosis. After continuous treatment with goserelin and 6 cycles of chemotherapy via docetaxel, his PSA levels dropped to 1 µg/l. Despite treatment, the patient progressed 5 months later, undergoing a palliative transurethral resection (TUR). Eventually, liver metastasis developed 5 months after TUR, and the patient passed away 1 year after initial diagnosis [55]. PDO‐xenografts (PDOXs) were obtained by subcutaneous injection of dissociated PDO cells in NSG male mice in the context of another study and biobanked for further applications. PDOX tumors were then dissociated and cultured as organoids (PDOXOs) as described by Dolgos et al. (2025) [56]. Early‐passage PDOXOs were maintained in prostate organoid medium (OGM; detailed composition provided in [30] and Supporting Information file), enzymatically dissociated into small aggregates, and seeded directly onto pre‐established osteoblastic/osteoclastic niches. For assessment of cell viability in hydroxyapatite, P20‐11 cells were seeded onto naked Engipore, and cultured for 10 days in OCM or OGM. The PDOXO cells were seeded in matrigel in the same medium conditions as controls. Their viability was assessed via CellTiter‐Glo Cell Viability Luminescence Assay, according to the manufacturer's instructions.

PCa bone metastasis samples (Bone Met #1, castration‐resistant prostate cancer, Bone Met #2 under androgen‐deprivation therapy, Bone Met #3, hormone‐sensitive prostate cancer; all derived from biopsy of vertebra adenocarcinoma) were used for immunofluorescence controls and were obtained under approval by the Ethics Committee of Northwestern and Central Switzerland (EKNZ 37/13) with informed patient consent in the context of previous studies [30, 57].

### 5.3 3D Niche Engineering and Co‐Culture With PCa Cells

#### 5.3.1 3D Niche Engineering and Osteogenic Differentiation

Osteoblastic niches (OBN) and osteoblastic–osteoclastic niches (OBCN) were established under static conditions, as illustrated in Figure 1A. Briefly, a cell suspension of 5 × 10^5^ BM‐MSCs in 60 µL of CM was pipetted onto 3D scaffolds, which were then placed in low‐attachment 12‐well plates. Commercially available 3D scaffolds were used for engineering the niches as previously described (Engipore, Finceramica) [25, 26, 27, 58, 59]. Briefly, the scaffolds consisted of disks 8 mm in diameter and 4 mm in height made of hydroxyapatite with ∼ 5 wt.% beta‐tricalcium phosphate. The internal structure simulates trabecular bone, being comprised an interconnected network of pores giving it total porosity of 83% ± 3% and the following pore‐size distribution: 22% <100 µm, 32% 100–200 µm, 40% 200–500 µm, 6% >500 µm. After 40 min of incubation at 37°C to allow for cell attachment, scaffolds were cultured in PM for 7 days, with a medium change on day 3. Suppliers and catalog numbers are provided in the Supporting Information file. Scaffolds were then cultured in OM for 21 days to promote osteogenic differentiation, with medium changes twice weekly.

#### 5.3.2 Seeding of Osteoclastic Precursors and Dual‐Lineage Niche Formation

To generate OBCNs, OM was removed, and scaffolds were inverted to expose the scaffold face that had not been in contact with the MSC suspension. This approach ensured access to the inner pores, which are otherwise occluded by the dense MSC layer, thereby improving CD14^+^ cell infiltration. A suspension of 1 x 10^6^ freshly isolated CD14^+^ monocytes in 20 µL of OCM was pipetted onto the exposed surface of each scaffold and incubated for 40 min at 37°C. Constructs were then cultured in OCM for 14 days. OBNs underwent the same handling procedure but without CD14^+^ cell seeding. Three distinct PBMC donors were used as a source of monocytes, one used for each different group of OBCNs and OBNs obtained by a different source of BM‐MSCs.

#### 5.3.3 PCa Cell Seeding and Experimental Timeline

For PCa co‐culture, transduced mEmerald‐LNCaP, mCherry‐PC3 cells, or organoid‐derived cells (P20‐11) were seeded by pipetting 1 × 10^6^ cells in 20 µL of OCM directly onto each niche after removing the medium. Following a 40‐min incubation at 37°C, scaffolds were cultured in OCM until analysis, which was performed at day 7 (timepoint 1, TP1) and day 14 (timepoint 2, TP2) after CD14^+^ addition. The co‐culture duration with PCa cells was limited to 10 days after tumor cell seeding. We chose this timeline based on the observed in vitro lifespan culture of human monocyte‐derived osteoclasts in previous works [25, 60, 61].

### 5.4 Cell Isolation From 3D Constructs

Niche constructs were retrieved from culture, rinsed once in PBS, and quartered using a sterile scalpel. Scaffold fragments were transferred into 1.5 mL tubes and enzymatically digested in two sequential steps: first with 0.15% Type II Collagenase at 37°C for 45 min with shaking at 400 rpm, followed by 0.25% Trypsin‐EDTA for 15 min under the same conditions. This combination enabled efficient matrix degradation and single‐cell release.

The resulting cell suspension was diluted 1:4 in FACS buffer (PBS supplemented with 0.5% FBS and 2 mm EDTA), centrifuged at 400 × g for 5 min, and resuspended in fresh buffer. To ensure a single‐cell suspension, samples were filtered twice through 70 µm cell strainers. Cell preparations were used for flow cytometry and other downstream applications. Reagent sources are listed in the Supporting Information File.

### 5.5 Sorting of mEmerald‐LNCaP and mCherry‐PC3 Cells From Bone Niche Constructs

Cell suspensions obtained from digested scaffolds at the endpoint of culture were subjected to fluorescence‐activated cell sorting (FACS) to isolate mEmerald^+^ LNCaP and mCherry^+^ PC3 cells from the total niche‐derived population (Figure 2A). Gating strategies included forward and side scatter area (FSC‐A vs. SSC‐A), and singlet discrimination using FSC‐A vs. FSC‐H and SSC‐A vs. SSC‐W with fluorescence‐based selection of the respective reporter‐positive tumor populations. Control constructs without fluorescently tagged tumor cells were processed in parallel using identical gating and sorting parameters to control for potential sorting‐induced effects on downstream analyzes.

### 5.6 RNA Extraction, cDNA Synthesis, and qRT‐PCR Analysis

Total RNA from sorted cell populations was extracted using either the RNeasy Mini Kit or the RNeasy Micro Kit, depending on cell yield, according to the manufacturer's instructions. RNA concentration and purity were assessed using a NanoDrop One spectrophotometer. Complementary DNA (cDNA) was synthesized via reverse transcription using SuperScript III Reverse Transcriptase.

Quantitative real‐time PCR (qRT‐PCR) was performed using a ViiA 7 Real‐Time PCR System and TaqMan gene expression assays targeting integrin‐binding sialoprotein (*IBSP*), osteocalcin (*BGLAP*), bone morphogenetic protein 2 (*BMP2*), alkaline phosphatase (*ALPL*), osteopontin (*OPN*), tartrate‐resistant acid phosphatase (*TRAP*), and osteonectin (*SPARC*). *GAPDH* was used as the housekeeping gene. Relative gene expression levels were calculated using the 2^−ΔCT^ method. Suppliers and catalog numbers are provided in the Supporting Information file.

### 5.7 Whole‐Mount Immunofluorescence Staining and Confocal Microscopy

Scaffolds were removed from culture and fixed overnight at 4°C in 4% formaldehyde. Samples were then permeabilized and blocked for 6 h at room temperature in PBS containing 0.4% Triton X‐100, 2% bovine serum albumin (BSA), and 5% goat serum. Fixed constructs were then incubated at 4°C for 70 h with primary antibodies against cytokeratin‐8 (CK8) and tartrate‐resistant acid phosphatase (TRAP), 1:200 in blocking solution. Samples were washed in PBS and incubated overnight at 4°C with secondary antibodies, Goat Anti‐Mouse 546 and Goat Anti‐Rabbit 647 each at 1:200 in PBS with 0.4% Triton X‐100 and 2% BSA. DAPI was used as a nuclear counterstain. Suppliers and catalog numbers are provided in the Supporting Information file. Samples were mounted on 35 mm imaging dishes and imaged using a Nikon Ti2‐E inverted microscope equipped with an AxR point‐scanning confocal unit and controlled via Nikon NIS software.

### 5.8 Histological Processing, Immunohistochemistry, and Immunofluorescence Staining

Following culture, the engineered niches were rinsed in PBS and fixed overnight in 4% formaldehyde. Samples were embedded in HistoGel and decalcified by incubation in 15% EDTA at 37°C for two weeks. Decalcified samples were embedded in paraffin and sectioned for histological analysis. Engineered tissue sections were deparaffinized, rehydrated, and stained with hematoxylin and eosin (H&E) using a standardized protocol on an automated stainer (Epredia Gemini AS). We used standard indirect immunoperoxidase procedures for IHC staining of bone sialoprotein (IBSP), osteocalcin (OCN), osteopontin (OPN), osteonectin (ON), Tartrate Resistant Acid Phosphatase (TRAP), and E‐Cadherin (E‐Cad) on the Ventana BenchMark ULTRA automated immunostainer. Whole‐slide images were acquired using a slide scanner (40x objective, NA 0.75).

For immunofluorescence, after deparaffinization, antigen retrieval was performed by incubating slides in pH 6 citrate buffer at 96°C for 15 min. Sections were then permeabilized in PBS containing 0.4% Triton X‐100 (PBS‐T) and blocked in PBS‐T with 10% goat serum. Primary antibodies for cytokeratin‐8 (CK8), OCN, OPN, and TRAP were incubated overnight at 1:200 in PBS‐T with 1% goat serum. Sections were then incubated with secondary antibodies, Goat Anti‐Rabbit 488, and Goat Anti‐Mouse 647, diluted 1:200 in PBS with 1% goat serum. DAPI was added for nuclear counterstaining. Images were acquired using a Nikon Ti2 widefield fluorescence microscope equipped with a Nikon DS‐Ri2 camera and controlled via NIS Elements AR software. Suppliers and catalog numbers are provided in the Supporting Information file.

Qualitative histological observations, including the morphological identification of specific cell types, was consistently supported by an expert pathologist.

### 5.9 Quantification of PCa Organoid Aggregates

At both timepoints, niches co‐cultured with the PCa organoid line P20‐11 were fixed and stained by whole‐mount immunofluorescence for cytokeratin‐8 (CK8), then counterstained with DAPI. Confocal z‐stacks were acquired at low magnification, and we performed volumetric image analysis to quantify the number and size of CK8^+^ tumor aggregates. The data represent three independent OBCN co‐cultures. Full image acquisition and analysis parameters are detailed in the Section S1.

### 5.10 Statistics

Statistical analysis of qRT‐PCR data was performed using GraphPad Prism (version 10.4.2). Group comparisons were conducted using Welch's *t*‐test to account for unequal variances. All tests were two‐tailed. *P*‐values were plotted as numerical values. Tumor volumes measured at 4 (TP1) and 10 days (TP2) were log_2_‐transformed, and comparisons between the two time points were performed using the non‐parametric Mann–Whitney U test. Experimental groups were designed such that they included n = 4 technical replicates. In the case of qRT‐PCR, outlier samples that did not show amplified signals were removed from analysis, leaving some groups with an n = 3.

## Funding

This project received funding from the European Union's Horizon 2020 research and innovation programme under the Marie Skłodowska‑Curie Innovative Training Network Sinergia (Grant Agreement No 860715). Financial support was also provided by the Department of Surgery at the University Hospital Basel (PMC Platform).

## Conflicts of Interest

The authors declare no conflicts of interest.

## Supporting information




**Supporting File 1**: adhm71334‐sup‐0001‐SuppMat.pdf.

**Supporting File 2**: adhm71334‐sup‐0002‐MovieS1.mp4.

**Supporting File 3**: adhm71334‐sup‐0003‐MovieS2.mp4.

## Data Availability

The data supporting the findings of this study are available within the article and its Supplementary Information files or from the corresponding author upon reasonable request.

## References

[1] World Health Ogranisation , “Cancer Today,” (accessed July 22, 2025), https://gco.iarc.who.int/today/.

[2] R. Raychaudhuri , D. W. Lin , and R. B. Montgomery , “Prostate Cancer: A Review,” JAMA 333 (2025): 1433–1446, 10.1001/jama.2025.0228. 40063046

[3] S. Casimiro , T. A. Guise , and J. Chirgwin , “The Critical Role of the Bone Microenvironment in Cancer Metastases,” Molecular and Cellular Endocrinology 310 (2009): 71–81, 10.1016/j.mce.2009.07.004. 19616059

[4] J.‐J. Body , S. Casimiro , and L. Costa , “Targeting Bone Metastases in Prostate Cancer: Improving Clinical Outcome,” Nature Reviews Urology 12 (2015): 340–356, 10.1038/nrurol.2015.90. 26119830

[5] R. B. Berish , A. N. Ali , P. G. Telmer , J. A. Ronald , and H. S. Leong , “Translational Models of Prostate Cancer Bone Metastasis,” Nature Reviews Urology 15 (2018): 403–421, 10.1038/s41585-018-0020-2. 29769644

[6] D. G. Hackam and D. A. Redelmeier , “Translation of Research Evidence From Animals to Humans,” JAMA 296 (2006): 1727, 10.1001/jama.296.14.1731. 17032985

[7] F. Salamanna , D. Contartese , M. Maglio , and M. Fini , “A Systematic Review on in Vitro 3D Bone Metastases Models: A New Horizon to Recapitulate the Native Clinical Scenario?,” Oncotarget 7 (2016): 44803–44820, 10.18632/oncotarget.8394. 27027241 PMC5190136

[8] N. Cristini , M. Tavakoli , M. Sanati , and S. A. Yavari , “Exploring Bone‐Tumor Interactions Through 3D *in Vitro* Models: Implications for Primary and Metastatic Cancers,” Journal of Bone Oncology 53 (2025): 100698, 10.1016/j.jbo.2025.100698. 40606222 PMC12219387

[9] C. Sánchez‐de‐Diego , R. C. Yada , N. Sethakorn , et al., “Engineering the Bone Metastatic Prostate Cancer Niche Through a Microphysiological System to Report Patient‐Specific Treatment Response,” Communications Biology 8 (2025): 961, 10.1038/s42003-025-08384-2. 40596459 PMC12218145

[10] N. Bock , A. Shokoohmand , T. Kryza , et al., “Engineering Osteoblastic Metastases to Delineate the Adaptive Response of Androgen‐Deprived Prostate Cancer in the Bone Metastatic Microenvironment,” Bone Research 7 (2019): 13, 10.1038/s41413-019-0049-8. 31044095 PMC6486620

[11] A. Bessot , F. M. Savi , J. Gunter , et al., “Humanized in Vivo Bone Tissue Engineering: in Vitro Preculture Conditions Control the Structural, Cellular, and Matrix Composition of Humanized Bone Organs,” Advanced Healthcare Materials 14 (2025): 2401939, 10.1002/adhm.202401939. 39444080 PMC11729988

[12] K. S. Koeneman , F. Yeung , and L. W. K. Chung , “Osteomimetic Properties of Prostate Cancer Cells: A Hypothesis Supporting the Predilection of Prostate Cancer Metastasis and Growth in the Bone Environment,” The Prostate 39 (1999): 246–261, 10.1002/(SICI)1097-0045(19990601)39:4<246::AID-PROS5>3.0.CO;2-U. 10344214

[13] S.‐Y. Sung , C.‐L. Hsieh , A. Law , et al., “Coevolution of Prostate Cancer and Bone Stroma in Three‐Dimensional Coculture: Implications for Cancer Growth and Metastasis,” Cancer Research 68 (2008): 9996–10003, 10.1158/0008-5472.CAN-08-2492. 19047182 PMC3105756

[14] S. Sieh , A. A. Lubik , J. A. Clements , C. C. Nelson , and D. W. Hutmacher , “Interactions Between Human Osteoblasts and Prostate Cancer Cells in a Novel 3D in Vitro Model,” Organogenesis 6 (2010): 181–188, 10.4161/org.6.3.12041. 21197221 PMC2946051

[15] N. Bock , T. Kryza , A. Shokoohmand , et al., “In Vitro Engineering of a Bone Metastases Model Allows for Study of the Effects of Antiandrogen Therapies in Advanced Prostate Cancer,” Science Advances 7 (2021): abg2564, 10.1126/sciadv.abg2564. PMC8245033 34193425

[16] M. Wang , F. Xia , Y. Wei , and X. Wei , “Molecular Mechanisms and Clinical Management of Cancer Bone Metastasis,” Bone Research 8 (2020): 30, 10.1038/s41413-020-00105-1. 32793401 PMC7391760

[17] H. Yonou , A. Ochiai , M. Goya , et al., “Intraosseous Growth of Human Prostate Cancer in Implanted Adult Human Bone: Relationship of Prostate Cancer Cells to Osteoclasts in Osteoblastic Metastatic Lesions,” The Prostate 58 (2004): 406–413, 10.1002/pros.10349. 14968441

[18] I. Roato , P. D'Amelio , E. Gorassini , et al., “Osteoclasts Are Active in Bone Forming Metastases of Prostate Cancer Patients,” PLoS ONE 3 (2008): 3627, 10.1371/journal.pone.0003627. PMC2574033 18978943

[19] X. Chen , X. Zhi , J. Wang , and J. Su , “RANKL Signaling in Bone Marrow Mesenchymal Stem Cells Negatively Regulates Osteoblastic Bone Formation,” Bone Research 6 (2018): 34, 10.1038/s41413-018-0035-6. 30510839 PMC6255918

[20] W. Cai , Y. Huo , Y. Liu , et al., “Biomechanics in Bone Regeneration and Mechanobiology in Osteoblasts: Fundamental Concepts and Recent Progress,” EngMedicine 2 (2025): 100057, 10.1016/j.engmed.2025.100057.

[21] K. Urano , Y. Tanaka , T. Tominari , et al., “The Stiffness and Collagen Control Differentiation of Osteoclasts With an Altered Expression of c‐Src in Podosome,” Biochemical and Biophysical Research Communications 704 (2024): 149636, 10.1016/j.bbrc.2024.149636. 38402724

[22] A. Matsugaki , T. Harada , Y. Kimura , A. Sekita , and T. Nakano , “Dynamic Collision Behavior Between Osteoblasts and Tumor Cells Regulates the Disordered Arrangement of Collagen Fiber/Apatite Crystals in Metastasized Bone,” International Journal of Molecular Sciences 19 (2018): 3474, 10.3390/ijms19113474. 30400633 PMC6274720

[23] F. Salamanna , V. Borsari , S. Brogini , et al., “An in Vitro 3D Bone Metastasis Model by Using a Human Bone Tissue Culture and Human Sex‐Related Cancer Cells,” Oncotarget 7 (2016): 76966–76983, 10.18632/oncotarget.12763. 27765913 PMC5363563

[24] S. Marino , R. T. Bishop , G. Carrasco , J. G. Logan , B. Li , and A. I. Idris , “Pharmacological Inhibition of NFκB Reduces Prostate Cancer Related Osteoclastogenesis In Vitro and Osteolysis Ex Vivo,” Calcified Tissue International 105 (2019): 193–204, 10.1007/s00223-019-00538-9. 30929064

[25] A. Papadimitropoulos , A. Scherberich , S. Güven , et al., “A 3D In Vitro Bone Organ Model Using Human Progenitor Cells,” European Cells and Materials 21 (2011): 445–458, 10.22203/ecm.v021a33. 21604244

[26] P. E. Bourgine , T. Klein , A. M. Paczulla , et al., “In Vitro Biomimetic Engineering of a Human Hematopoietic Niche With Functional Properties,” Proceedings of the National Academy of Sciences 115 (2018): E5688–E5695, 10.1073/pnas.1805440115. PMC6016789 29866839

[27] G. Born , M. Nikolova , A. Scherberich , B. Treutlein , A. García‐García , and I. Martin , “Engineering of Fully Humanized and Vascularized 3D Bone Marrow Niches Sustaining Undifferentiated Human Cord Blood Hematopoietic Stem and Progenitor Cells,” Journal of Tissue Engineering 12 (2021): 20417314211044855, 10.1177/20417314211044855. 34616539 PMC8488506

[28] S. Mondal , S. Park , J. Choi , et al., “Hydroxyapatite: A Journey From Biomaterials to Advanced Functional Materials,” Advances in Colloid and Interface Science 321 (2023): 103013, 10.1016/j.cis.2023.103013. 37839281

[29] N. Ribeiro , S. R. Sousa , R. A. Brekken , and F. J. Monteiro , “Role of SPARC in Bone Remodeling and Cancer‐Related Bone Metastasis,” Journal of Cellular Biochemistry 115 (2014): 17–26, 10.1002/jcb.24649. 24038053

[30] R. Servant , M. Garioni , T. Vlajnic , et al., “Prostate Cancer Patient‐Derived Organoids: Detailed Outcome From a Prospective Cohort of 81 Clinical Specimens,” The Journal of Pathology 254 (2021): 543–555, 10.1002/path.5698. 33934365 PMC8361965

[31] R. Parmentier , R. Dolgos , R. Servant , et al., “A Modular Patient‐Derived Organoid–Xenograft Platform Reveals Molecular and Clinical Trajectories of Prostate Cancer Progression,” bioRxiv (2026): 697006, 10.64898/2026.01.05.697006.

[32] J.‐M. Kim , C. Lin , Z. Stavre , M. B. Greenblatt , and J.‐H. Shim , “Osteoblast‐Osteoclast Communication and Bone Homeostasis,” Cells 9 (2020): 2073, 10.3390/cells9092073. 32927921 PMC7564526

[33] S.‐K. Lee , J. Kalinowski , S. Jastrzebski , and J. A. Lorenzo , “1,25 (OH)_2_ Vitamin D_3_‐Stimulated Osteoclast Formation in Spleen‐Osteoblast Cocultures Is Mediated in Part by Enhanced IL‐1α and Receptor Activator of NF‐κB Ligand Production in Osteoblasts1,” Journal of Immunology 169 (2002): 2374–2380, 10.4049/jimmunol.169.5.2374. 12193704

[34] S. Kitazawa , K. Kajimoto , T. Kondo , and R. Kitazawa , “Vitamin D_3_ Supports Osteoclastogenesis via Functional Vitamin D Response Element of Human RANKL Gene Promoter,” Journal of Cellular Biochemistry 89 (2003): 771–777, 10.1002/jcb.10567. 12858342

[35] M. A. Chellaiah , N. Kizer , R. Biswas , et al., “Osteopontin Deficiency Produces Osteoclast Dysfunction due to Reduced CD44 Surface Expression,” Molecular Biology of the Cell 14 (2003): 173–189, 10.1091/mbc.e02-06-0354. 12529435 PMC140236

[36] J. Luukkonen , M. Hilli , M. Nakamura , et al., “Osteoclasts Secrete Osteopontin into Resorption Lacunae During Bone Resorption,” Histochemistry and Cell Biology 151 (2019): 475–487, 10.1007/s00418-019-01770-y. 30637455 PMC6542781

[37] J. H. Tae and I. H. Chang , “Animal Models of Bone Metastatic Prostate Cancer,” Investigative and Clinical Urology 64 (2023): 219–228, 10.4111/icu.20230026. 37341002 PMC10172043

[38] N. Rucci and A. Teti , “Osteomimicry: How Tumor Cells Try to Deceive the Bone,” Frontiers in Bioscience 2 (2010): 907–915, 10.2741/S110. 20515833

[39] X. Pang , R. Xie , Z. Zhang , Q. Liu , S. Wu , and Y. Cui , “Identification of SPP1 as an Extracellular Matrix Signature for Metastatic Castration‐Resistant Prostate Cancer,” Frontiers in Oncology 9 (2019): 924, 10.3389/fonc.2019.00924. 31620371 PMC6760472

[40] L. M. Adams , M. J. Warburton , and A. R. Hayman , “Human Breast Cancer Cell Lines and Tissues Express Tartrate‐Resistant Acid Phosphatase (TRAP),” Cell Biology International 31 (2007): 191–195, 10.1016/j.cellbi.2006.09.022. 17088078

[41] S. Zenger , W. He , B. Ek‐Rylander , et al., “Differential Expression of Tartrate‐Resistant Acid Phosphatase Isoforms 5a and 5b by Tumor and Stromal Cells in Human Metastatic Bone Disease,” Clinical & Experimental Metastasis 28 (2011): 65–73, 10.1007/s10585-010-9358-4. 20967488

[42] J. H. Kim and N. Kim , “Regulation of NFATc1 in Osteoclast Differentiation,” Journal of Bone Metabolism 21 (2014): 233–241, 10.11005/jbm.2014.21.4.233. 25489571 PMC4255043

[43] A. P. Armstrong , R. E. Miller , J. C. Jones , J. Zhang , E. T. Keller , and W. C. Dougall , “RANKL Acts Directly on RANK‐Expressing Prostate Tumor Cells and Mediates Migration and Expression of Tumor Metastasis Genes,” The Prostate 68 (2008): 92–104, 10.1002/pros.20678. 18008334

[44] M. Malinen , E. A. Niskanen , M. U. Kaikkonen , and J. J. Palvimo , “Crosstalk Between Androgen and Pro‐Inflammatory Signaling Remodels Androgen Receptor and NF‐κB Cistrome to Reprogram the Prostate Cancer Cell Transcriptome,” Nucleic Acids Research 45 (2017): 619–630, 10.1093/nar/gkw855. 27672034 PMC5314794

[45] X. Pang , K. Gong , X. Zhang , S. Wu , Y. Cui , and B.‐Z. Qian , “Osteopontin as a Multifaceted Driver of Bone Metastasis and Drug Resistance,” Pharmacological Research 144 (2019): 235–244, 10.1016/j.phrs.2019.04.030. 31028902

[46] A. Buskin , E. Scott , R. Nelson , et al., “Engineering Prostate Cancer In Vitro: What Does It Take?,” Oncogene 42 (2023): 2417–2427, 10.1038/s41388-023-02776-6. 37438470 PMC10403358

[47] S. Choudhary , P. Ramasundaram , E. Dziopa , et al., “Human Ex Vivo 3D Bone Model Recapitulates Osteocyte Response to Metastatic Prostate Cancer,” Scientific Reports 8 (2018): 17975, 10.1038/s41598-018-36424-x. 30568232 PMC6299475

[48] A. Shokoohmand , J. Ren , J. Baldwin , et al., “Microenvironment Engineering of Osteoblastic Bone Metastases Reveals Osteomimicry of Patient‐Derived Prostate Cancer Xenografts,” Biomaterials 220 (2019): 119402, 10.1016/j.biomaterials.2019.119402. 31400612

[49] C. Paindelli , V. Parietti , S. Barrios , et al., “Bone Mimetic Environments Support Engineering, Propagation, and Analysis of Therapeutic Response of Patient‐Derived Cells, Ex Vivo and In Vivo,” Acta Biomaterialia 178 (2024): 83–92, 10.1016/j.actbio.2024.02.025. 38387748 PMC12016311

[50] S. H. Park , E. T. Keller , and Y. Shiozawa , “Bone Marrow Microenvironment as a Regulator and Therapeutic Target for Prostate Cancer Bone Metastasis,” Calcified Tissue International 102 (2018): 152–162, 10.1007/s00223-017-0350-8. 29094177 PMC5807175

[51] M. E. Sowder and R. W. Johnson , “Bone as a Preferential Site for Metastasis,” JBMR Plus 3 (2019): 10126, 10.1002/jbm4.10126. PMC6419612 30918918

[52] E. C. Kabak , S. L. Foo , M. Rafaeva , I. Martin , and M. Bentires‐Alj , “Microenvironmental Regulation of Dormancy in Breast Cancer Metastasis: “An Ally That Changes Allegiances,”,” A Guide to Breast Cancer Research: From Cellular Heterogeneity and Molecular Mechanisms to Therapy, ed. T. Sørlie and R. B. Clarke (Springer Nature, 2025), 373–395, 10.1007/978-3-031-70875-6_18. 39821034

[53] M. M. McDonald , W. H. Khoo , P. Y. Ng , et al., “Osteoclasts Recycle via Osteomorphs During RANKL‐Stimulated Bone Resorption,” Cell 184 (2021): 1330–1347.e13, 10.1016/j.cell.2021.02.002. 33636130 PMC7938889

[54] N. Sadr , B. E. Pippenger , A. Scherberich , et al., “Enhancing the Biological Performance of Synthetic Polymeric Materials by Decoration With Engineered, Decellularized Extracellular Matrix,” Biomaterials 33 (2012): 5085–5093, 10.1016/j.biomaterials.2012.03.082. 22510434

[55] R. Parmentier , R. Dolgos , R. Servant , et al., “A Modular Patient‐Derived Organoid–Xenograft Platform Reveals Molecular and Clinical Trajectories of Prostate Cancer Progression,” bioRxiv (2026): 697006, 10.64898/2026.01.05.697006.

[56] R. Dolgos , R. Parmentier , J. Wang , et al., “Single‐Cell Analysis Uncovers Preserved Prostate Cancer Lineages and Universally Altered Pathways in Matrigel‐Free Patient‐Derived Organoids,” Cell Reports 44 (2025): 116352, 10.1016/j.celrep.2025.116352. 41037396

[57] R. Dolgos , R. Parmentier , J. Wang , et al., “ECM‐Free Patient‐Derived Organoids Preserve Diverse Prostate Cancer Lineages and Uncover in Vitro‐Enriched Cell Types,” bioRxiv (2024): 618617, 10.1101/2024.10.16.618617.

[58] A. Braccini , D. Wendt , C. Jaquiery , et al., “Three‐Dimensional Perfusion Culture of Human Bone Marrow Cells and Generation of Osteoinductive Grafts,” Stem Cells 23 (2005): 1066–1072, 10.1634/stemcells.2005-0002. 16002780

[59] Q. Li , M. T. Nikolova , G. Zhang , et al., “Macro‐Scale, Scaffold‐Assisted Model of the Human Bone Marrow Endosteal Niche Using hiPSC‐Vascularized Osteoblastic Organoids,” Cell Stem Cell 32 (2025): 1941–1958, 10.1016/j.stem.2025.10.009. 41260216

[60] K. Fujisaki , N. Tanabe , N. Suzuki , et al., “Receptor Activator of NF‐κB Ligand Induces the Expression of Carbonic Anhydrase II, Cathepsin K, and Matrix Metalloproteinase‐9 in Osteoclast Precursor RAW264.7 Cells,” Life Sciences 80 (2007): 1311–1318, 10.1016/j.lfs.2006.12.037. 17306833

[61] E. Rossi , E. Mracsko , A. Papadimitropoulos , et al., “An In Vitro Bone Model to Investigate the Role of Triggering Receptor Expressed on Myeloid Cells‐2 in Bone Homeostasis,” Tissue Engineering Part C: Methods 24 (2018): 391–398, 10.1089/ten.tec.2018.0061. 29897015

