# Supplementary material for: A Fully Human Engineered Bone Niche With Endogenous Osteoclastogenesis Reveals Osteoclast‐Dependent Osteomimicry in Prostate Cancer Cells
Source: Adv Healthc Mater. 2026 Jun 11;15(26):e05893. doi: 10.1002/adhm.202505893 (PMC13356505; doi:10.1002/adhm.202505893)
Supplement: Supplementary file 1 — Supporting File 1: adhm71334‐sup‐0001‐SuppMat.pdf. [file ADHM-15-0-s003.pdf]

1. Supplementary Methods

1.1. Lentiviral labeling of PCa cell lines

LNCaP and PC3 cells were stably labeled with mEmerald and mCherry fluorescent reporters, respectively, using third-generation lentiviral vectors based on the pLVX backbone and carrying a neomycin resistance cassette. Lentiviral particles were produced in Lenti-X 293T cells using standard packaging plasmids and Lipofectamine 2000. Cells were transduced at a multiplicity of infection (MOI) of 2 in the presence of polybrene, and selected with G418 for 10 days. Stable expression was confirmed by fluorescence microscopy before co-culture. Suppliers and catalog numbers are provided in the Supplementary Information file.

1.2. FACS gating and instrument parameters

Sorting was performed using either a CytoFLEX SRT (Beckman Coulter) or FACSAria III (BD Biosciences), depending on instrument availability. mEmerald^{+} LNCaP cells were detected using the green channel (B525/40 on the CytoFLEX SRT; 488–530/30 on the FACSAria III), and mCherry^{+} PC3 cells were detected using the red channel (Y610/20 on the CytoFLEX SRT; 561–616/23 on the FACSAria III). Initial gating was based on FSC-A vs. SSC-A, followed by singlet discrimination using FSC-A vs. FSC-H and SSC-A vs. SSC-W. Fluorescence compensation and gating thresholds were established using single-color controls and non-fluorescent negative samples processed identically to the experimental groups.

1.3. Whole-mount immunofluorescence confocal acquisition parameters

Samples were mounted in 35 mm imaging dishes (Ibidi) and imaged using a Nikon Ti2-E inverted microscope equipped with an AxR point-scanning confocal unit and NIS-Elements software. Fluorescence excitation was performed using 405, 488, 561, and 640 nm lasers, with emission detection windows at 429–474, 499–525, 571–605, and 662–737 nm, respectively. Images were acquired in 12-bit format using resonant scanning mode with 16× line averaging, 2048 × 2048 pixel resolution, and a 1 Airy unit pinhole (16 µm at 640 nm). Z-stacks were collected across 50–600 µm depths using AutoSignal.ai to optimize laser power and detector gain. Step intervals were 11.6 µm (4×), 2.1 µm (10×), 1.0 µm (20×), and 0.3 µm (40×) objectives.

1.4. Image acquisition and volumetric quantification of organoid aggregates

Confocal z-stacks were acquired on a Nikon Ti2-E microscope equipped with an AxR confocal scanning unit, using a 4× objective. Z-stacks consisted of 27 to 52 optical sections with a z-step of 11.6 µm.

Image stacks were converted to volumetric .ims format using Imaris File Converter 10.1.0 and analyzed in Imaris 10.1.0 (Bitplane) using the Measurement Pro module. Surfaces were generated from the CK8 channel via absolute intensity thresholding, using the default surface reconstruction algorithm. Objects with a volume smaller than 8×10^3 μm^3 were excluded to eliminate background signal and debris. A total number of n=3 constructs were imaged per timepoint, for whichthe number and volume of $CK8^+$ tumor aggregates were quantified (ranging from 115 to 339 observations depending on the construct) and exported for downstream statistical analysis.

1.5. Scanning electron microscopy (SEM)

At each timepoint, bone niche constructs were fixed overnight in 2% glutaraldehyde prepared in 0.1 M sodium cacodylate buffer (pH 7.4). Samples were dehydrated through a graded ethanol series (30%, 50%, 70%, 90%, and 100%) with 15-minute incubations at each step. Dehydrated constructs were processed using a critical point dryer (Leica EM CPD300) and mounted on aluminum stubs using carbon adhesive. Samples were sputter-coated with a 20 nm layer of gold using a Leica EM ACE600 coater. SEM images were acquired using a ZEISS Gemini 2 field-emission scanning electron microscope operating at 5 kV accelerating voltage and 200 pA probe current. Catalog numbers and reagent details are listed in the SI File.

2. Supplementary Figures

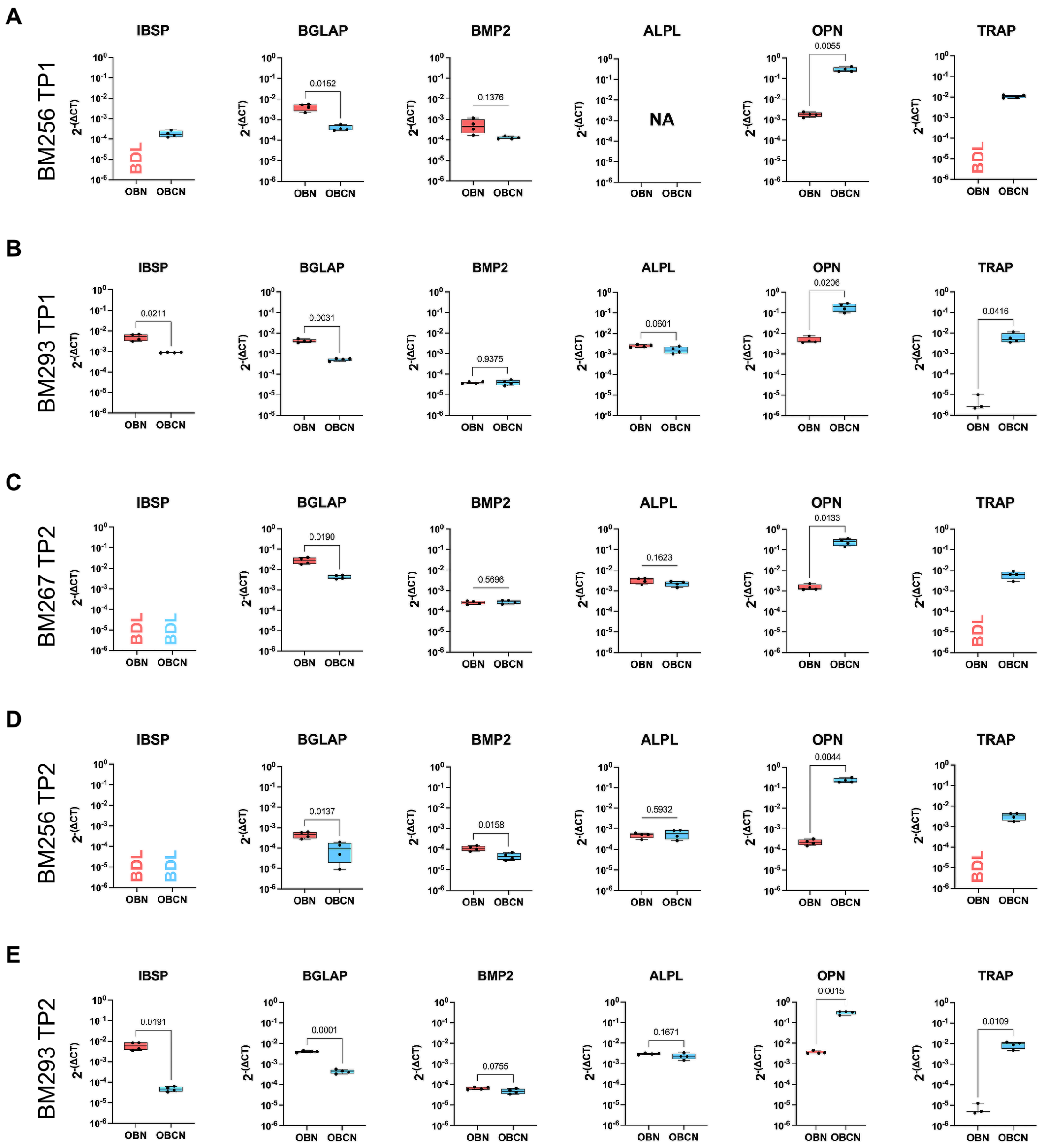

Supplementary Figure 1 - Osteoblastic and osteoclastic marker expression compared between OBNs (red) and OBCNs (blue) by qRT-PCR analysis. The analysis was performed for markers IBSP, BGLAP, BMP2, ALPL, OPN, and TRAP. Analysis was done for the following donors and conditions: (A) donor BM256 at TP1, (B) BM293 at TP1, (C) BM267 at TP2, (D) BM256 at TP2, (E) BM293 at TP2. Expression

level was normalized to GAPDH and calculated using the $2^{-\Delta CT}$ method. Each graph represents data from a singular donor, at the first analysis timepoint, with experimental replicates per group plotted as circles on each graph (up to n=4). Comparisons were done via Welch t-test, with p-value shown in numeric format. Undetectable levels of gene expression were marked as BDL (below detection level), while ALPL for BM256 at TP1 could not be assessed and was marked as NA (not available).

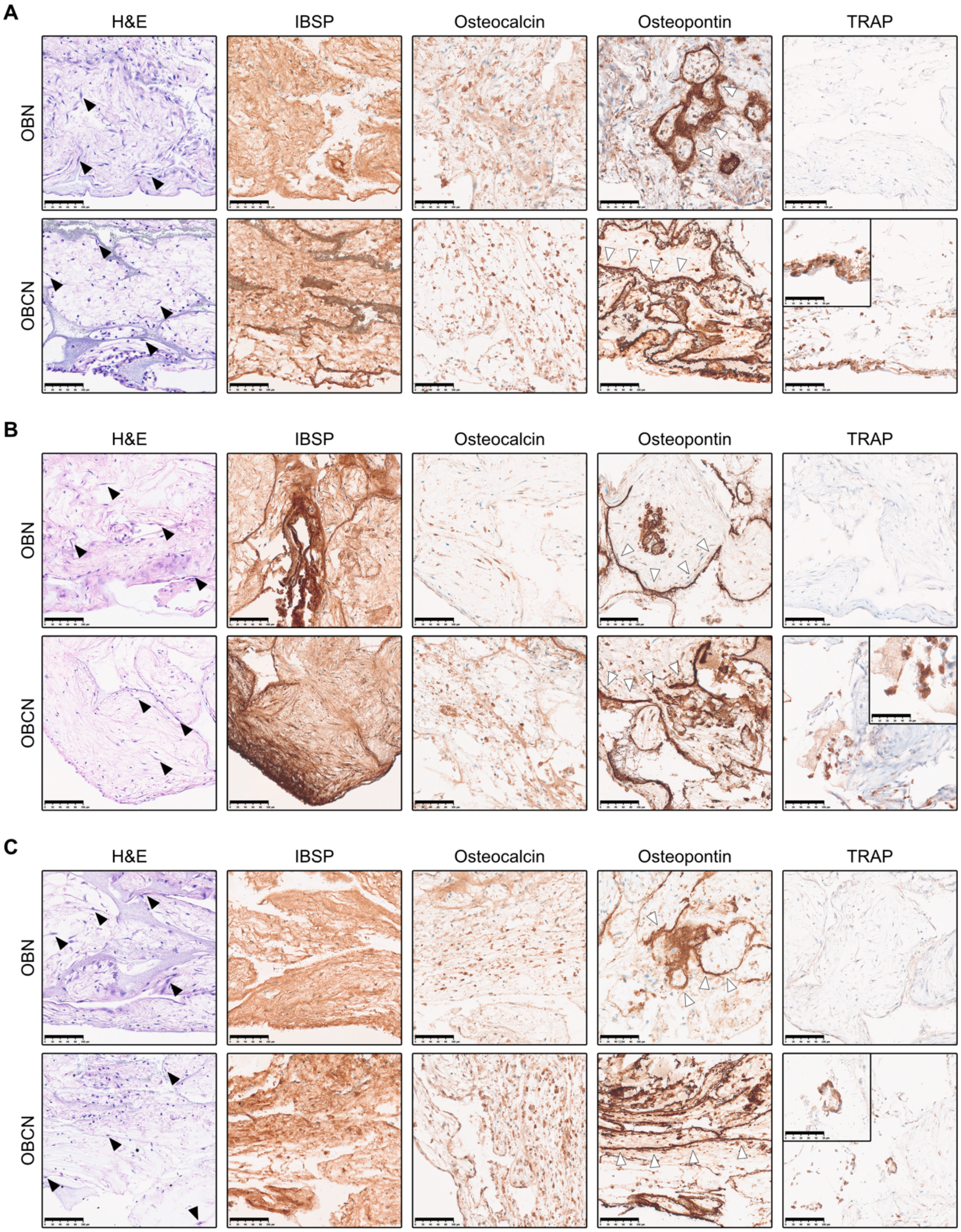

Supplementary Figure 2 - Immunohistochemistry (IHC) imaging of osteoblastic and osteoclastic markers in OBNs and OBCNs. Niches were imaged via H&E staining and IHC for IBSP, osteocalcin, osteopontin, and TRAP (Scale bars: 100 μm). Imaging was

done for donors (A) BM256 at TP1, (B) BM267 at TP2, and (C) BM256 at TP2. Black arrowheads in H&E images indicate cells with fusiform morphology, indicative of osteoblasts. White arrowheads in osteopontin images indicate areas adjacent to scaffold-derived hydroxyapatite. Detailed inset shown for TRAP to highlight multi-nucleated TRAP+ cells (Scale bar: 50 μm).

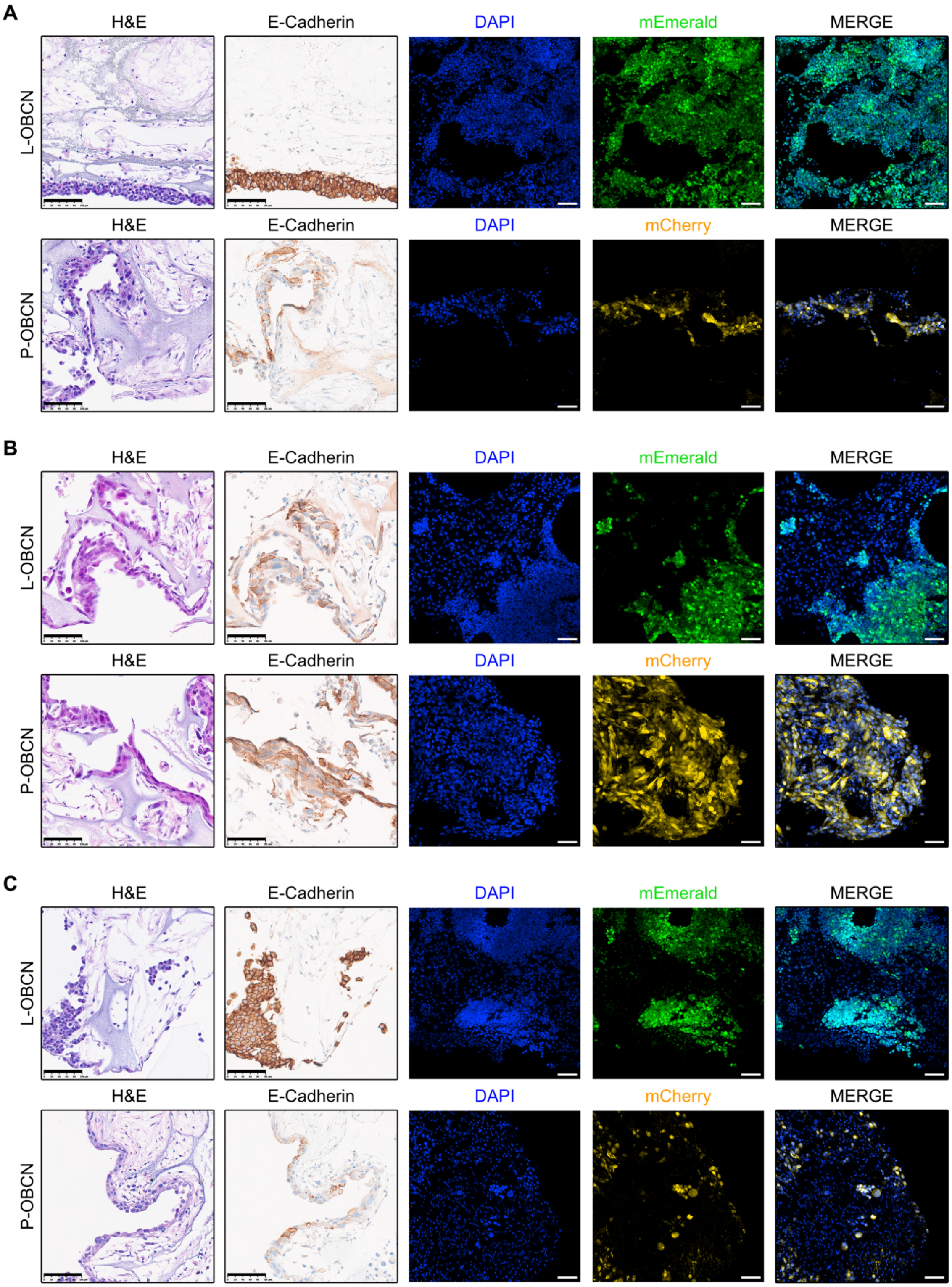

Supplementary Figure 3 - Histological and immunofluorescence analysis of LNCaP and PC3 cell engraftment in OBCNs. Representative images from L-OBCN and P-

OBCN niches derived from: (A) donor BM256 at TP1, (B) BM267 at TP2, and (C) BM256 at TP2. Samples were analyzed by H&E staining, E-Cadherin IHC, and whole-mount IF. Fluorescent channels include DAPI (nuclei), mEmerald (LNCaP), and mCherry (PC3). Individual channels and merged images are shown. Scale bars: 100 μm.

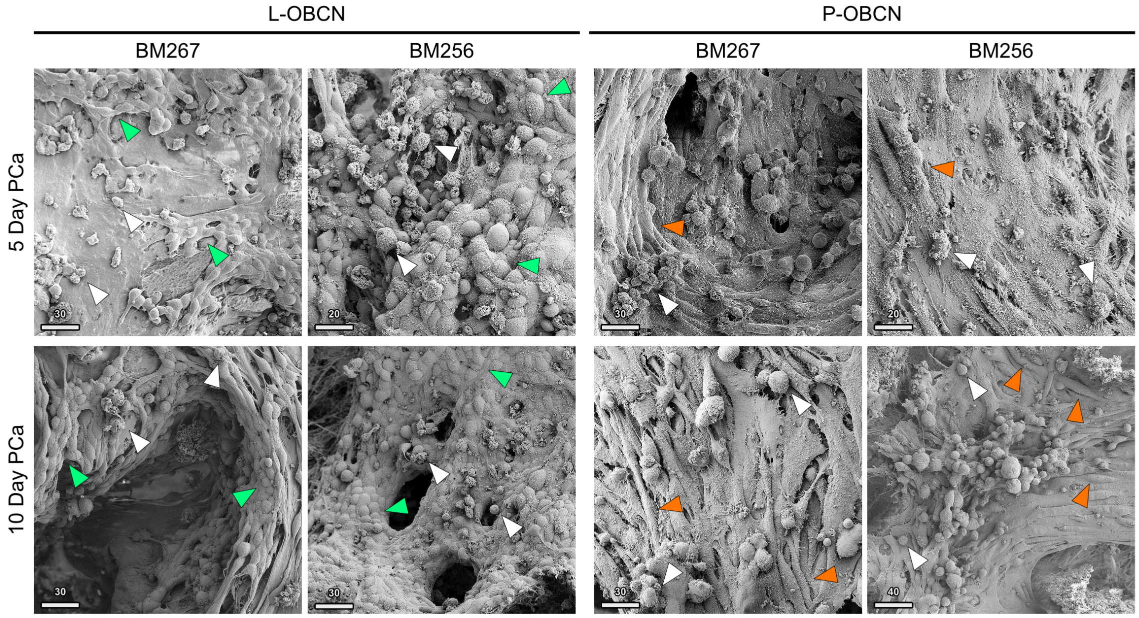

Supplementary Figure 4 - Scanning electron microscopy (SEM) of prostate cancer cell engraftment in the niche. SEM images of L-OBCNs and P-OBCNs generated from donors BM267 and BM256, acquired at day 4 (TP1) and day 10 (TP2) of co-culture with PCa cells. LNCaP and PC3 cells are indicated by green and orange arrowheads, respectively. White arrowheads highlight monocyte-derived cells within the niche. Scale bars: 20–40 µm.

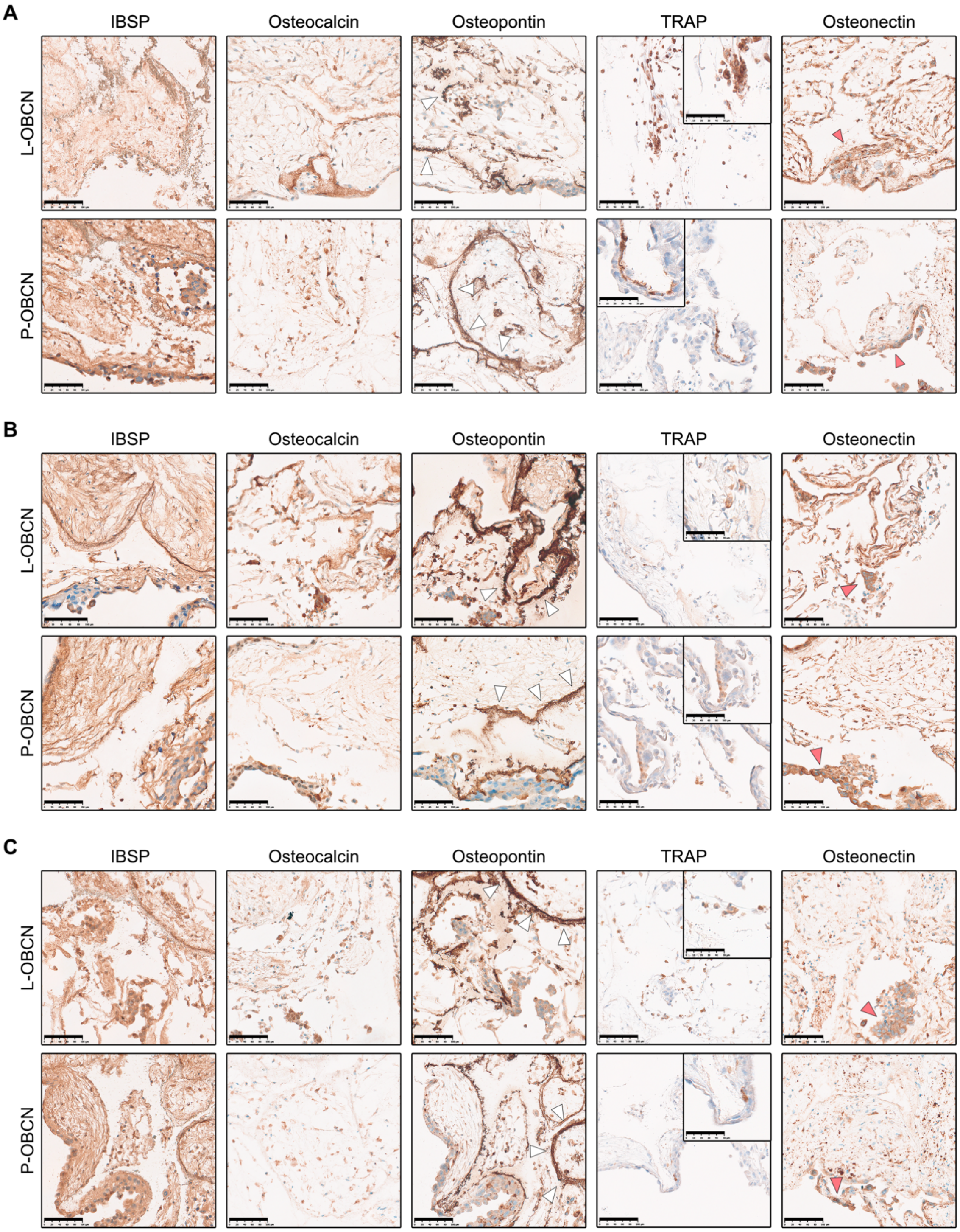

Supplementary Figure 5 - Histological assessment of bone-related markers in PCa co-cultured OBCNs. Representative IHC staining for key bone microenvironment markers (IBSP, osteocalcin, osteopontin, TRAP, and osteonectin) in L-OBCNs and P-

OBCNs derived from: (A) BM256 at TP1, (B) BM267 at TP2, and (C) BM256 at TP2. White arrowheads in osteopontin panels highlight areas adjacent to scaffold-derived hydroxyapatite. Red arrowheads indicate PCa cells positive for osteonectin. High-magnification insets in TRAP panels show multinucleated $TRAP^+$ cells. Scale bars: 100 µm (main panels), 50 µm (TRAP insets).

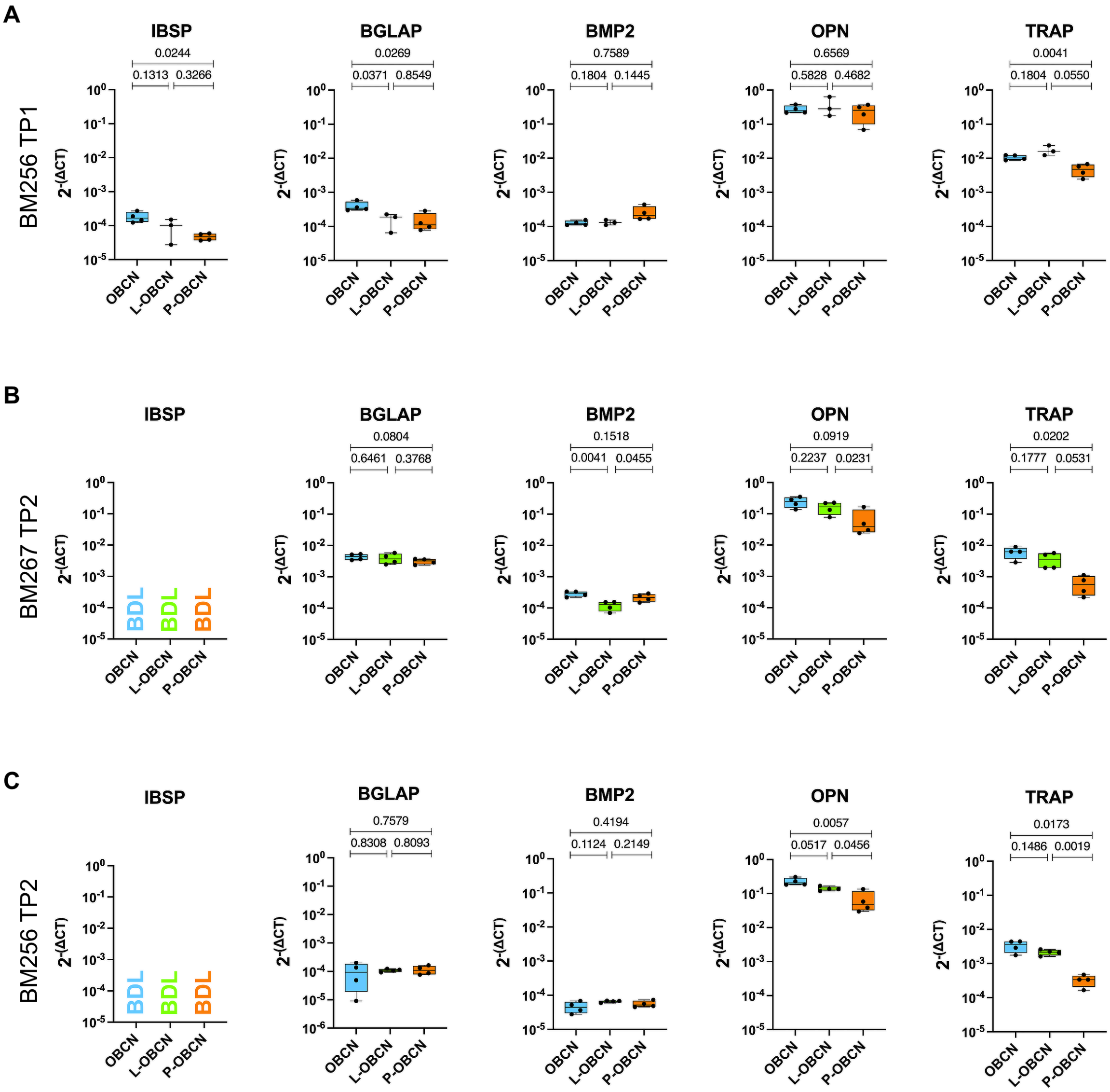

Supplementary Figure 6 - Gene expression analysis of key bone-related markers in non-tumor niche cells sorted from OBCNs, L-OBCNs, and P-OBCNs. qRT-PCR analysis of osteoblastic and osteoclastic markers IBSP, BGLAP, BMP2, OPN, and TRAP on osteoblasts and osteoclasts retrieved from the OBCNs after sorting, for (A) donor BM256 at TP1, (B) BM267 at TP2, and (C) BM256 at TP2. Comparisons are drawn between cells extracted from OBCNs without PCa (blue), L-OBCNs (green), and P-OBCNs (orange). Comparisons were done via Welch t-test, with numerical p-value shown. Experimental replicates per group plotted as circles on each graph (up to n=4).

Samples with undetectable gene expression have been marked with BDL (below detection level).

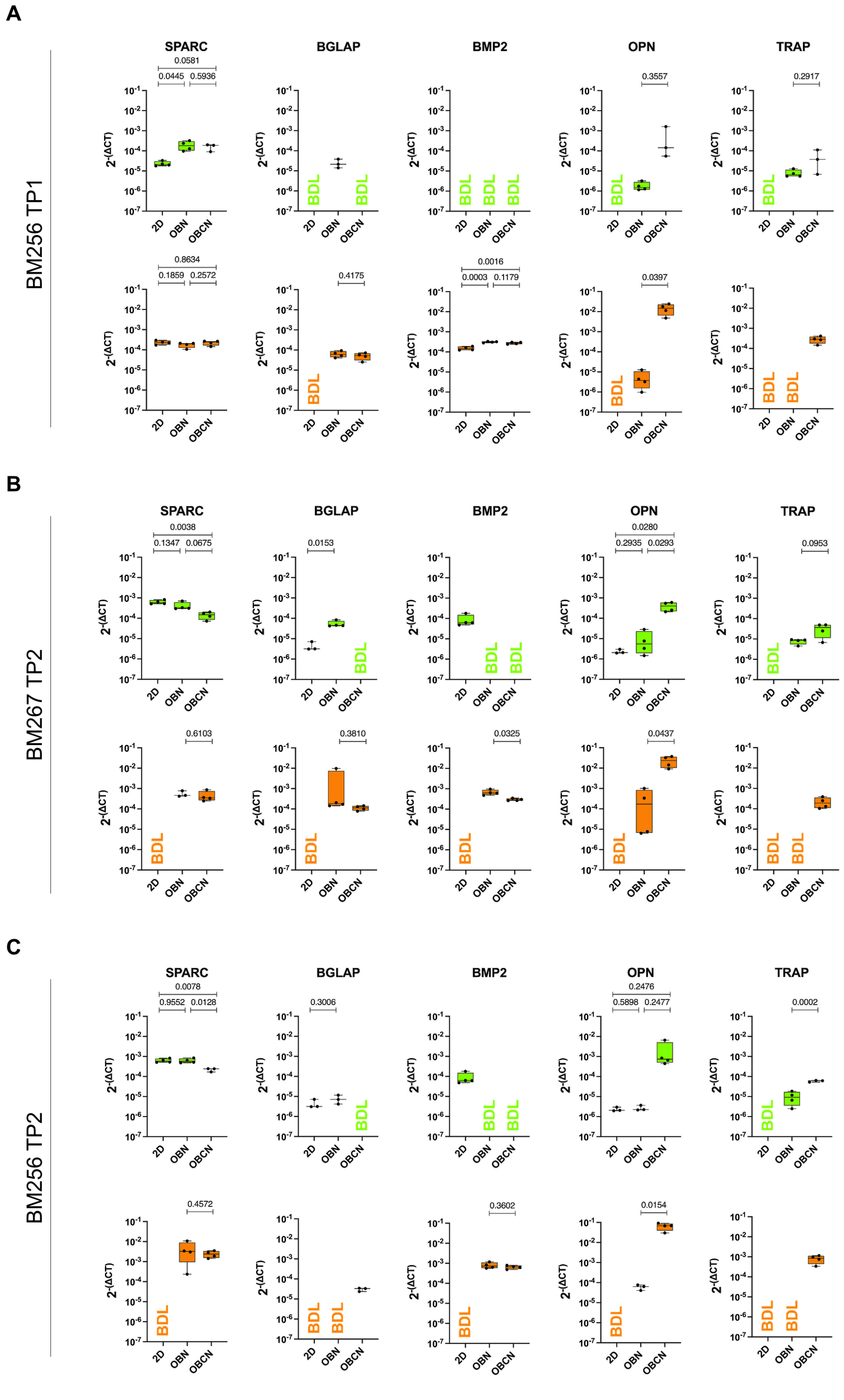
A
BM256 TP1
SPARC
BGLAP
BMP2
OPN
TRAP
0.0581
0.0445
0.5936
0.3557
0.2917
0.8634
0.1859
0.2572
0.4175
0.0016
0.0003
0.1179
0.0397
2-(ΔCT)
BDL
2D
OBN
OBCN
B
BM267 TP2
SPARC
BGLAP
BMP2
OPN
TRAP
0.0038
0.1347
0.0675
0.0153
0.0280
0.2935
0.0293
0.0953
0.6103
0.3810
0.0325
0.0437
C
BM256 TP2
SPARC
BGLAP
BMP2
OPN
TRAP
0.0078
0.9552
0.0128
0.3006
0.2476
0.5898
0.2477
0.0002
0.4572
0.3602
0.0154

Supplementary Figure 7 - Analysis of gene expression of bone-related markers in PCa cell lines extracted from L-OBCNs and P-OBCNs compared to their 2D-grown counterparts. qRT-PCR analysis of osteoblastic and osteoclastic markers SPARC, BGLAP, BMP2, OPN, and TRAP on PCa cells extracted from L-OBNs and L-OBCNs (green) or P-OBNs and P-OBCNs (orange) for (A) BM256 at TP1, (B) BM267 at TP2, and (C) BM256 at TP2. Comparisons are drawn between cells co-cultured in the OBCNs and the same kind of PCa line grown in 2D under the same medium conditions. Comparisons were done via Welch t-test, with p-value shown in number format. Experimental replicates per group plotted as circles on each graph (up to n=4). Samples with undetectable gene expression have been marked with BDL (below detection level).

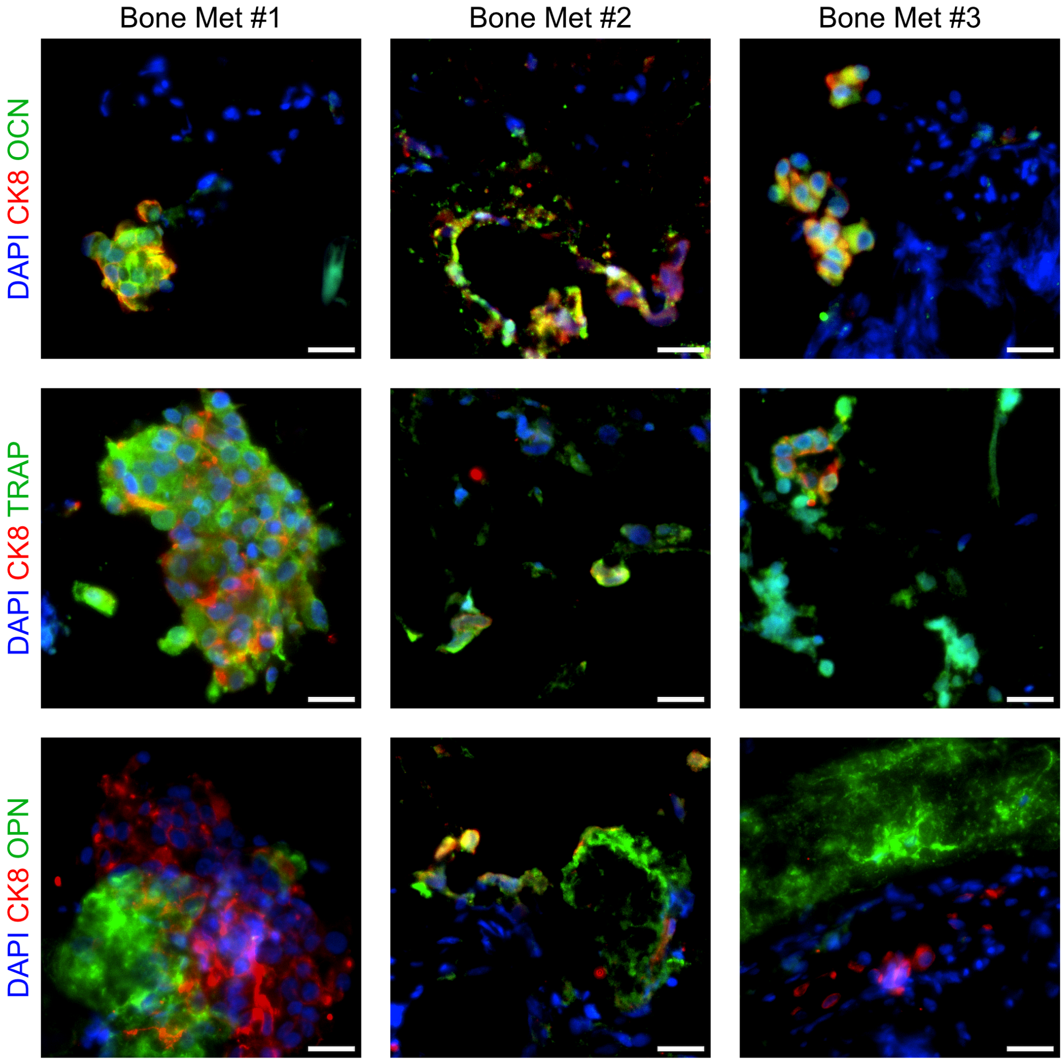

Supplementary Figure 8 - Evidence of osteomimetic behaviour in patient-derived PCa-to-bone metastasis samples. Immunofluorescence images showing expression of bone-related markers osteocalcin, TRAP, and osteopontin (green) alongside localisation of cells expressing Cytokeratin 8 (red) from patient-derived control samples from PCa bone metastasis (Scale bar: 20 μm).

3. Reagents and Instruments List

The section number for each table refers to the section in the manuscript.

**2.1 Cell Culture Media**			
**Reagent / Component Name**	**Supplier**	**Catalog Number**	**Concentration**
Minimum Essential Medium Alpha (aMEM)	Gibco	22571	Pure
Fetal Bovine Serum (FBS)	Gibco	A5256701	10%
GlutaMAX	Gibco	35050	1%
Penicillin-Streptomycin	Gibco	15140	1%
HEPES	Gibco	15630	1%
Sodium Pyruvate	Gibco	11360	1%
Fibroblast Growth Factor 2 (FGF2)	R&D Systems	233-FB	5 ng/mL
Ascorbic Acid	Sigma-Aldrich	A8960	0.1 mM
Dexamethasone	Sigma-Aldrich	D2915	10 nM
Beta-Glycerophosphate	Sigma-Aldrich	G9422	10 mM
1,25-Dihydroxyvitamin D3	Tocris	2551	10 nM
Macrophage Colony-Stimulating Factor (M-CSF)	Gibco	AF-300-25	25 ng/mL

**2.2 Cell Source and Cell Expansion**			
**Reagent / Component Name**	**Supplier**	**Catalog Number**	
Ficoll-Paque PLUS	Cytiva	17144002	
CD14 MicroBeads	Miltenyi Biotec	130-050-201	
LS Columns	Miltenyi Biotec	130-042	
QuadroMACS Separator	Miltenyi Biotec	130-091-051	
CellTiter-Glo	Promega	G9681	
**Cell Line Name**	**Supplier**	**Catalog Number**	
LNCaP prostate cancer cell line	DMSZ	ACC256	
PC3 prostate cancer cell line	ATCC	CRL-1435	

Prostate organoid medium (OGM) components:	Supplier	Catalog Number	Concentration
AdDMEM-F12	Gibco	12634010	Pure
Glutamax	Gibco	35050061	1%
Hepes	Gibco	15630056	1%
Pen/Strep	BioConcept	4-01F00-H	1%
Primocin	InvivoGen	ant-pm-1	100 μg/ml
B27	Life Technologies	17504044	1X
Nicotinamide	Sigma-Aldrich	N0636-100g	10 mm
N-Acetyl-l-cysteine	Sigma-Aldrich	A9165-25g	1.25 mm
A83-01	Tocris	2939	500 nm
FGF-10	Peprotech	100-26-25UG	10 ng/ml
FGF-2	Peprotech	100-18B-50UG	5 ng/ml
EGF	Peprotech	AF-100-15-100UG	5 ng/ml
Noggin	Peprotech	120-10C-50UG	100 or 50 ng/ml
Rspondin 1	R&D	4645-RS-100	500 or 250 ng/ml
Prostaglandin E2	Tocris	2296	1 μm
SB202190	Sigma-Aldrich	S7076-5MG	10 μm
Y-27632 dihydrochloride	Abmole Bioscience	M1817	10 μm
(Dihydro)testosterone (5α-androstan-17β-ol-3-one)	Sigma-Aldrich	10300-5G-F	1 or 0.1 nm

**2.3 3D Niche Co-culture**			
**Reagent / Component Name**	**Supplier**	**Catalog Number**	
Porous hydroxyapatite scaffold (8×4 mm)	Finceramica	ENGI-PROD-01	
Low-attachment 12-well plates	Sarstedt	4021721	

**2.4 Cell Isolation from the 3D Constructs**			
**Reagent / Component Name**	**Supplier**	**Catalog Number**	**Concentration**
Type II Collagenase	Worthington	LS004176	0.15%

Thermomixer C	Eppendorf	5382000015	—
Trypsin-EDTA (0.25%)	Gibco	25200072	—
70 µm Cell Strainers	Corning	352350	—

**2.5 Sorting of mEmerald-LNCaP and mCherry-OC3 cells from OBNs and OBCNs**			
**Reagent / Component Name**	**Supplier**	**Catalog Number**	
CytoFLEX SRT	Beckman Coulter		
FACSAria III	BD Biosciences		

**2.6 RNA extraction, cDNA synthesis, and qRT-PCR analysis**			
**Reagent / Equipment Name**	**Supplier**	**Catalog Number**	
RNeasy Mini Kit	Qiagen	74106	
RNeasy Micro Kit	Qiagen	74004	
NanoDrop One Spectrophotometer	Thermo Fisher	ND-ONE-W	
SuperScript III Reverse Transcriptase	Thermo Fisher	18080093	
ViiA 7 Real-Time PCR System	Thermo Fisher	—	
TaqMan Assay – IBSP	Thermo Fisher	Hs00173720_m1	
TaqMan Assay – BGLAP	Thermo Fisher	Hs01587814_g1	
TaqMan Assay – BMP2	Thermo Fisher	Hs00154192_m1	
TaqMan Assay – ALPL	Thermo Fisher	Hs01029144_m1	
TaqMan Assay – OPN	Thermo Fisher	Hs00959010_m1	
TaqMan Assay – TRAP	Thermo Fisher	Hs00356261_m1	
TaqMan Assay – SPARC	Thermo Fisher	Hs00234160_m1	
TaqMan Assay – GAPDH (endogenous control)	Thermo Fisher	Hs02758991_g1	

**2.7 Whole-mount immunofluorescence staining and confocal microscopy**			
**Reagent / Equipment Name**	**Supplier**	**Catalog Number**	**Concentrat**

			ion
Formaldehyde 4%	Thermo Fisher	28908	4%
Triton X-100	Sigma-Aldrich	93443	0.40%
Bovine Serum Albumin (BSA)	Sigma-Aldrich	A9647	2%
Goat Serum	Gibco	16210064	1%, 5%, or 10%
Anti-Cytokeratin-8 (CK8) Antibody	Biolegend	904804	1 : 200
Anti-TRAP Antibody	Abcam	AB185716	1 : 200
Goat Anti-Mouse 546 (Secondary)	Invitrogen	A11030	1 : 200
Goat Anti-Rabbit 647 (Secondary)	Invitrogen	A21244	1 : 200
DAPI	BD Biosciences	564907	1 mg/mL
Imaging Dishes (35 mm)	Ibidi	81218-200	—
Ti2-E Inverted Microscope	Nikon	—	—
AxR Point-Scanning Confocal Unit	Nikon	—	—
NIS Elements Software	Nikon	—	—

**2.8 Histological processing, immunohistochemistry, and immunofluorescence staining**			
**Reagent / Equipment Name**	**Supplier**	**Catalog Number**	**Concentration**
HistoGel	Epredia	HG-4000-012	Pure
EDTA	Sigma-Aldrich	E6635	15%
TPC-15 Trio	MEDITE Medical	—	—
Microtome	Thermo Scientific	—	—
SuperFrost Plus Glass Slides	Fisherbrand	—	—
Xylene (Xylol Histograde)	Biosystems	3410	Pure
Citrate Buffer pH 6 (50x)	quartett Q Retrieval	AR-001-0120	1 : 50
Triton X-100	Sigma-Aldrich	93443	0.40%
Goat Serum	Gibco	16210064	1%
Anti-Cytokeratin-8 (CK8) Antibody	Biolegend	904804	1 : 200
Anti-Osteocalcin (OCN) Antibody	Thermo Fisher	PA5-96529	1 : 200
Anti-E-Cadherin (E-CAD) Antibody	Ventana	760-4440	ready-to-use

Anti-TRAP Antibody	Bio BS	BSB5982	ready-to-use
Anti-Bone Sialoprotein (BSP)	Abcam	AB52128	1 : 100
Anti-Osteonectin (ON)	Thermo Fisher	33-5500	1 : 500
Anti-Osteopontin Antibody (OCN)	Proteintech	22952-1-AP	1 : 200
Goat Anti-Rabbit 488 (Secondary)	Invitrogen	A11034	1 : 200
Goat Anti-Mouse 647 (Secondary)	Invitrogen	A21236	1 : 200
DAPI	BD Biosciences	564907	1 mg/mL
Ventana BenchMark ULTRA	Roche Diagnostics	—	—
Fluoromount Aqueous Mounting Medium	Sigma-Aldrich	F4680	—
Gemini AS Automated Stainer	Epredia	—	—
NanoZoomer S60 Slide Scanner	Hamamatsu	—	—
Ti2 Widefield Fluorescence Microscope	Nikon	—	—
DS-Ri2 Camera	Nikon	—	—
NIS Elements AR Software (v5.21.03)	Nikon	—	—

**2.9 Quantification of PCa Organoids Aggregates**			
**Reagent / Software / Equipment Name**	**Supplier**	**Version / Catalog Number**	
Anti-Cytokeratin-8 (CK8) Antibody	Biolegend	904804	
DAPI	BD Biosciences	564907	
Nikon AxR Confocal Microscope	Nikon	—	
Imaris File Converter	Bitplane	v10.1.10	
Imaris with Measurement Pro Module	Bitplane	v10.1.10	

**2.10 Statistics**			
**Software / Method Name**	**Supplier**	**Version / Details**	
GraphPad Prism	GraphPad	v10.4.2	

**9.1 Lentiviral Labelling of PCa Cell Lines**			
**Reagent / Component Name**	**Supplier**	**Catalog Number**	**Concentration**
pLVX lentiviral vector backbone	Clontech/Takara Bio	632164	
Lenti-X 293T packaging cells	Clontech/Takara Bio	632180	
Packaging plasmid psPAX2	Addgene	12260	
Packaging plasmid pMD2.G	Addgene	12259	
Packaging plasmid pRSV-Rev (likely)	Addgene	12253	
Lipofectamine 2000	Thermo Fisher	11668027	
Polybrene	Sigma-Aldrich	H9268	8 µg/mL
G418 (Geneticin)	Roche Diagnostics	108321-42-2	250 µg/mL

**9.5 Scanning Electron Microscopy Imaging**			
**Reagent / Equipment Name**	**Supplier**	**Catalog Number**	**Concentration**
Glutaraldehyde	Thermo Scientific	A17876	2%
Sodium Cacodylate Buffer	Thermo Scientific	J62202.AE	0.1M
Critical Point Dryer	Tousimis	Autosamdri-815	—
Sputter Coater	LEICA	EM ACE600	—
SEM Microscope	ZEISS	Gemini 2	—

4. List of Abbreviations

Abbreviation	Full Term / Definition
ADT	Androgen Deprivation Therapy
ALPL	Alkaline Phosphatase (gene encoding alkaline phosphatase enzyme)
aMEM	Minimum Essential Medium Alpha (α-MEM)
AR	Androgen Receptor
BDL	Below Detection Level
BGLAP	Bone Gamma-Carboxyglutamate Protein (gene encoding Osteocalcin)
BM-MSC	Bone Marrow-derived Mesenchymal Stromal Cell
BMP2	Bone Morphogenetic Protein 2
BSA	Bovine Serum Albumin
cDNA	Complementary DNA
CK8	Cytokeratin-8
CM	Complete Medium
CRPC	Castration-Resistant Prostate Cancer
DAPI	4′,6-Diamidino-2-Phenylindole (nuclear counterstain)
E-Cad	E-Cadherin
EDTA	Ethylenediaminetetraacetic Acid
EKNZ	Ethics Committee of Northwestern and Central Switzerland (Ethikkommission Nordwest- und Zentralschweiz)
FACS	Fluorescence-Activated Cell Sorting

Abbreviation	Full Term / Definition
FBS	Fetal Bovine Serum
FFPE	Formalin-Fixed Paraffin-Embedded
FGF2	Fibroblast Growth Factor 2
FSC-A	Forward Scatter Area (flow cytometry parameter)
FSC-H	Forward Scatter Height (flow cytometry parameter)
GAPDH	Glyceraldehyde-3-Phosphate Dehydrogenase (housekeeping gene)
H&E	Hematoxylin and Eosin (histological stain)
HA	Hydroxyapatite
HSPC	Hormone-Sensitive Prostate Cancer
IBSP	Integrin-Binding Sialoprotein (gene encoding Bone Sialoprotein)
IF	Immunofluorescence
IHC	Immunohistochemistry
L-OBN / L-OBCN	LNCaP-seeded Osteoblastic Niche / LNCaP-seeded Osteoblastic-Osteoclastic Niche
LNCaP	Lymph Node Carcinoma of the Prostate (AR+, hormone-sensitive prostate cancer cell line)
MACS	Magnetic-Activated Cell Sorting
M-CSF	Macrophage Colony-Stimulating Factor
MOI	Multiplicity of Infection
mPCa	Metastatic Prostate Cancer
MSC	Mesenchymal Stromal Cell
NSG	NOD Scid Gamma (severely immunodeficient mouse strain)

Abbreviation	Full Term / Definition
OBN	Osteoblastic Niche (osteoblast-only construct; no osteoclasts)
OBCN	Osteoblastic-Osteoclastic Niche (dual-lineage construct)
OCM	Osteoclastic Medium
OCN	Osteocalcin
OGM	Organoid medium
OM	Osteoblastic (Osteogenic) Medium
ON	Osteonectin
OPN	Osteopontin
P-OBN / P-OBCN	PC3-seeded Osteoblastic Niche / PC3-seeded Osteoblastic-Osteoclastic Niche
PBS	Phosphate-Buffered Saline
PBS-T	Phosphate-Buffered Saline with Triton X-100 (permeabilization buffer)
PBMC	Peripheral Blood Mononuclear Cell
PC3	Prostate Cancer cell line 3 (AR−, castration-resistant, bone-derived)
PCa	Prostate Cancer
PDO	Patient-Derived Organoid
PDOXO	Patient-Derived Organoid-to-Xenograft-to-Organoid
PM	Proliferative Medium
qRT-PCR	Quantitative Reverse Transcription Polymerase Chain Reaction
RANKL	Receptor Activator of Nuclear Factor κB Ligand
RNA	Ribonucleic Acid
S1P	Sphingosine-1-Phosphate

Abbreviation	Full Term / Definition
SEM	Scanning Electron Microscopy
SEMA4D	Semaphorin 4D
SI	Supplementary Information
SM	Supplementary Methods
SPARC	Secreted Protein Acidic and Rich in Cysteine (gene encoding Osteonectin)
SPP1	Secreted Phosphoprotein 1 (gene encoding Osteopontin)
SRC	Swiss Red Cross
SSC-A	Side Scatter Area (flow cytometry parameter)
SSC-W	Side Scatter Width (flow cytometry parameter)
TP1	Time Point 1: 7 days post-osteoclast seeding, corresponding to 4 days post-PCa cell seeding
TP2	Time Point 2 14 days post-osteoclast seeding, corresponding to 10 days post-PCa cell seeding
TRAP	Tartrate-Resistant Acid Phosphatase
3D	Three-Dimensional
3R	Replacement, Reduction, Refinement
